# *Saccharomyces cerevisiae* Ski7 Is a GTP-Binding Protein Adopting the Characteristic Conformation of Active Translational GTPases

**DOI:** 10.1016/j.str.2015.04.018

**Published:** 2015-07-07

**Authors:** Eva Kowalinski, Anthony Schuller, Rachel Green, Elena Conti

**Affiliations:** 1 Department of Structural Cell Biology Department, Max Planck Institute of Biochemistry, Am Klopferspitz 18, 82152 Martinsried, Germany; 2 Howard Hughes Medical Institute, Department of Molecular Biology and Genetics, Johns Hopkins University School of Medicine, Baltimore, MD 21205, USA

## Abstract

Ski7 is a cofactor of the cytoplasmic exosome in budding yeast, functioning in both mRNA turnover and non-stop decay (NSD), a surveillance pathway that degrades faulty mRNAs lacking a stop codon. The C-terminal region of Ski7 (Ski7_C_) shares overall sequence similarity with the translational GTPase (trGTPase) Hbs1, but whether Ski7 has retained the properties of a trGTPase is unclear. Here, we report the high-resolution structures of Ski7_C_ bound to either intact guanosine triphosphate (GTP) or guanosine diphosphate-P_i_. The individual domains of Ski7_C_ adopt the conformation characteristic of active trGTPases. Furthermore, the nucleotide-binding site of Ski7_C_ shares similar features compared with active trGTPases, notably the presence of a characteristic monovalent cation. However, a suboptimal polar residue at the putative catalytic site and an unusual polar residue that interacts with the γ-phosphate of GTP distinguish Ski7 from other trGTPases, suggesting it might function rather as a GTP-binding protein than as a GTP-hydrolyzing enzyme.

## Highlights

• The Ski7 C-terminal region adopts the conformation of active translational GTPases
• Ski7 features the monovalent cation characteristic of active translational GTPases
• Ski7 has unusual features in the nucleotide-binding site and binds GTP stably
• Ski7 might have evolved as a pseudo-trGTPase with enhanced GTP-binding properties

## Graphical Abstract

**Figure:**
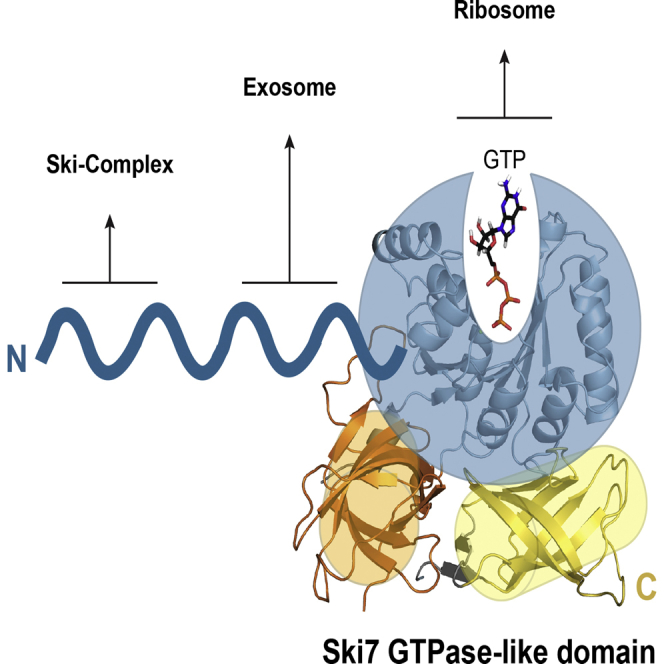

## Teaser

Kowalinski et al. report the crystal structure of the GTPase-like domain of yeast Ski7, a protein module involved in non-stop decay, a ribosome-dependent mRNA surveillance pathway. This study shows that Ski7 interacts stably with guanosine triphosphate (GTP) and adopts the overall structural features of ribosome-associated GTPases.

## Introduction

The decay of cytoplasmic mRNAs regulates the output of eukaryotic gene expression in terms of both quantity and quality. In general, mRNA turnover modulates the abundance of normal transcripts in the cell and thereby the quantity of the proteins they encode (reviewed in Garneau et al., 2007; Houseley and Tollervey, 2009; Parker, 2012). In addition, eukaryotic cells have evolved quality-control mechanisms that prompt the decay of defective mRNAs. These surveillance pathways act at translating ribosomes and recognize different types of defects, for example the absence of a stop codon (non-stop decay [NSD]), the presence of a premature stop codon (nonsense-mediated decay [NMD]) or the presence of features that cause abnormal stalling of the translation machinery (no-go decay [NGD]) (reviewed in Inada, 2013; Kervestin and Jacobson, 2012; Klauer and van Hoof, 2012; Lykke-Andersen and Bennett, 2014; Popp and Maquat, 2013; Schweingruber et al., 2013; Shoemaker and Green, 2012). In both normal and aberrant situations, enzymatic machineries eventually degrade the body of the mRNA either from the 5′ end (via Xrn1) or from the 3′ end (via the exosome) (reviewed in Garneau et al., 2007; Houseley and Tollervey, 2009).

The exosome core complex is a ten-subunit assembly endowed with processive 3′–5′ exoribonuclease activity (Mitchell et al., 1997; reviewed in Januszyk and Lima, 2014; Makino et al., 2013). In the cytoplasm, the exosome functions together with the Ski complex, a 4-subunit protein complex centered around an RNA helicase (Brown et al., 2000; Halbach et al., 2013). In *Saccharomyces cerevisiae*, the interaction between the exosome and the Ski complex is mediated by Ski7 (Araki et al., 2001; van Hoof et al., 2000). While the exosome and the Ski complexes are evolutionary conserved, Ski7 has long been thought to be unique to *Saccharomyces* species. Recently, however, it has been shown that *Lachancea kluyveri* expresses a Ski7 protein by alternative splicing of the *HBS1* gene (Atkinson et al., 2008; Marshall et al., 2013), raising the possibility that Ski7 might have a wider phylogenetic distribution than currently thought.

Ski7 is a multidomain protein. The N-terminal portion contains the regions that mediate binding to the exosome and Ski complexes (Araki et al., 2001) and is required for all exosome-mediated RNA decay pathways, including general mRNA turnover (van Hoof et al., 2002). The C-terminal part contains a GTPase domain and is required specifically in the NSD pathway (Frischmeyer et al., 2002; van Hoof et al., 2000). Interestingly in this context, the closest paralog of Ski7 is Hbs1, a translational GTPase (trGTPase) also involved in aberrant translation termination (reviewed in Hoshino, 2012). Hbs1 was originally implicated in NGD (Doma and Parker, 2006) and has recently been shown to participate in both NSD (Saito et al., 2013; Tsuboi et al., 2012) and the rescue of ribosomes arrested at the 3′ end of truncated mRNAs or stalled in the 3′ UTR (Guydosh and Green, 2014; Shoemaker et al., 2010).

Like all known GTPases, Hbs1 switches between an active GTP-bound conformation and an inactive guanosine diphosphate (GDP)-bound conformation (reviewed in Wittinghofer and Vetter, 2011). On its own, Hbs1 has negligible intrinsic GTPase activity (Shoemaker et al., 2010). However, the GTP hydrolysis reaction of Hbs1 is greatly stimulated by the presence of Dom34 and the ribosome, which together fulfill the function of a composite GTPase-activating protein (Shoemaker et al., 2010). In this sense, the Hbs1-Dom34 complex is analogous to the eRF1-eRF3 complex, which functions in translation termination at stop codons (reviewed in Zhou et al., 2012), and to the EFTu-aminoacyl-tRNA complex, which functions in translation elongation (reviewed in Voorhees and Ramakrishnan, 2013; Rodnina, 2009).

The overall similarities between Ski7 and its paralog Hbs1 are compelling. However, whether Ski7 is an active trGTPase is not clear, particularly because residues that are invariant in the catalytic site of canonical trGTPases are not conserved in Ski7. Furthermore, there is no cofactor known to associate with Ski7 that could fulfill a similar function to Dom34 or eRF1. In this work, we used structural approaches to shed light on the function of Ski7 and found that Ski7 can bind GTP and adopt the conformation of active GTP-bound trGTPases.

## Results and Discussion

### Structure Determination of the C-Terminal GTPase-Like Region of Ski7

We engineered a C-terminal fragment of *S. cerevisiae* Ski7 that encompasses the predicted GTPase-like region (residues 254–747, thereby referred to as Ski7_C_). Ski7_C_ was incubated with either GTP or GDP for crystallization trials. We obtained crystals of Ski7_C_ in the presence of GDP and magnesium ions using inorganic phosphate as precipitating agent. We solved the structure with a selenomethionine-based single-wavelength anomalous dispersion experiment and refined it at 2.2-Å resolution to *R*_free_ of 24.6%, *R*_work_ of 21.7%, and good stereochemistry (Table 1). The refined structure (referred to as Ski7_C_-GDP-P_i_) includes most of the Ski7_C_ polypeptide chain (with the exception of short disordered loop regions) and also includes GDP, a molecule of inorganic phosphate (P_i_) and an Mg^2+^ ion. Manganese was soaked into the crystal prior to data collection to substitute Mg^2+^ and to unambiguously identify the position of the divalent cation in the electron density using anomalous scattering (Figure S1A).

**Figure 1:**
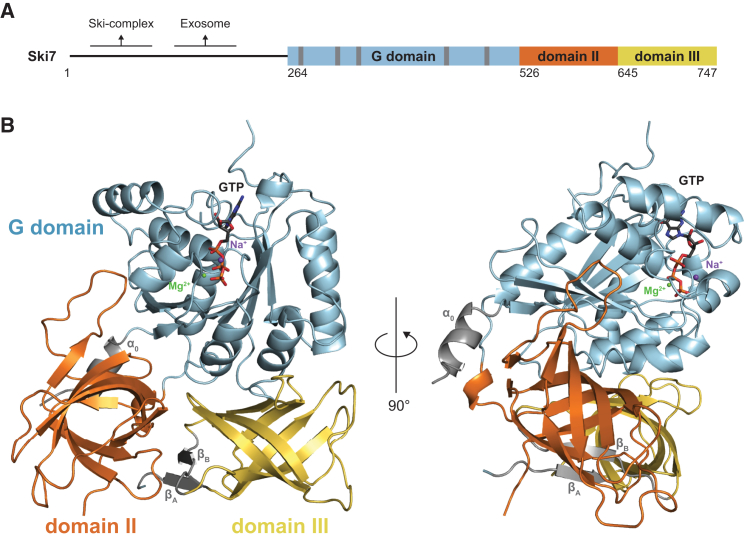
The Structure of the C-Terminal Domain of Ski7 Bound to GTP (A) Schematic representation of the domain arrangement of *S. cerevisiae* Ski7. Colored rectangles highlight the C-terminal domain of Ski7 (Ski7_C_) visualized in the structure reported here, with its three subdomains (G domain in cyan, domain II in orange, and domain III in yellow). The positions of the five conserved sequence motifs in the GTP-binding domain (G domain or domain I) are highlighted with gray bars. Regions of Ski7 that bind the Ski2-Ski3-Ski8 complex and the exosome complex are indicated. (B) The overall structure of Ski7_C_ bound to GTP shown in two orientations, related by a 90° anticlockwise rotation around a vertical axis. The structures are shown in cartoon representation with the G domain in cyan, domain II in orange, and domain III in yellow. GTP is shown in stick representations and the ions as spheres (green for Mg^2+^ and purple for Na^+^). Depicted in gray are the N-terminal α helix α_0_ as well as the β_A_ and β_B_ strands that are described in text. (See also Figure S1).

Using the same crystallization conditions, we also obtained crystals of Ski7_C_ in the presence of GTP. The structure of Ski7_C_-GTP was determined using the atomic coordinates of the protein chain from the Ski7_C_-GDP-P_i_ structure. The refinement showed the presence of well-defined electron density for GTP and an Mg^2+^ ion, and weaker density, which was interpreted as a monovalent metal ion (Figures 1B and S2A). The final model was refined to 2.3-Å resolution with *R*_free_ of 23.9%, *R*_work_ of 20.5%, and good stereochemistry (Table 1). Except for a loop approaching the sugar moiety of the nucleotide that is involved in a crystal contact in the Ski7_C_-GDP-P_i_ crystal (Figures S1B and S1D), the structure of Ski7_C_ is essentially identical in the Ski7_C_-GDP-P_i_ and Ski7_C_-GTP complexes, superposing with a root-mean-square deviation of 0.53 Å over all atoms (Figure S1C). The description of Ski7_C_ below thus refers to both structures, unless otherwise specified.

### The Ski7_C_ Structure Reveals a Domain Arrangement Typical of Active trGTPases

Ski7_C_ is organized in three domains (Figure 1A). Domain I (residues 264–518) adopts the αβ fold of GTP-binding domains (G domains), with a central six-stranded β sheet surrounded by α helices (Figure 1B). Domain II (residues 526–636) and domain III (residues 645–747) each adopt the structure of a closed β barrel (Figure 1B). The β barrels of Ski7_C_ are positioned side by side, with two antiparallel β strands (β_A_ and β_B_) wedged in between. The β_A_ and β_B_ strands are not adjacent in the sequence but form a small β sheet that interacts on one side with the barrel of domain II and on the other side with the barrel of domain III (Figure 1B). As observed for other GTPases of this family, domains II and III appear to form a single unit (Berchtold et al., 1993; Kjeldgaard et al., 1993).

Domains II and III both interact with the G domain (Figure 1B). The three domains interact intra-molecularly not only via their globular folds but also via extended segments. First, an α helix at the N terminus of the G domain (residues 254–264, α_0_) packs against domain II and against the linker that connects the two domains. Second, a loop connecting strands β_4_ and β_5_ of domain II interacts with the side of the G domain. The trGTPases Hbs1, eRF3, EFTu, and eIF5B share a similar sequel of globular domains (Figure S2A). Several crystal structures of trGTPases have been determined in complex with different GTP analogs and with cofactors (Figure S2B) (Chen et al., 2010; Kobayashi et al., 2010; Nissen et al., 1995; Preis et al., 2014). While the structures of the individual G domains or domains II-III of trGTPases superpose well, their relative orientation differs depending on the nucleotide state.

**Figure 2:**
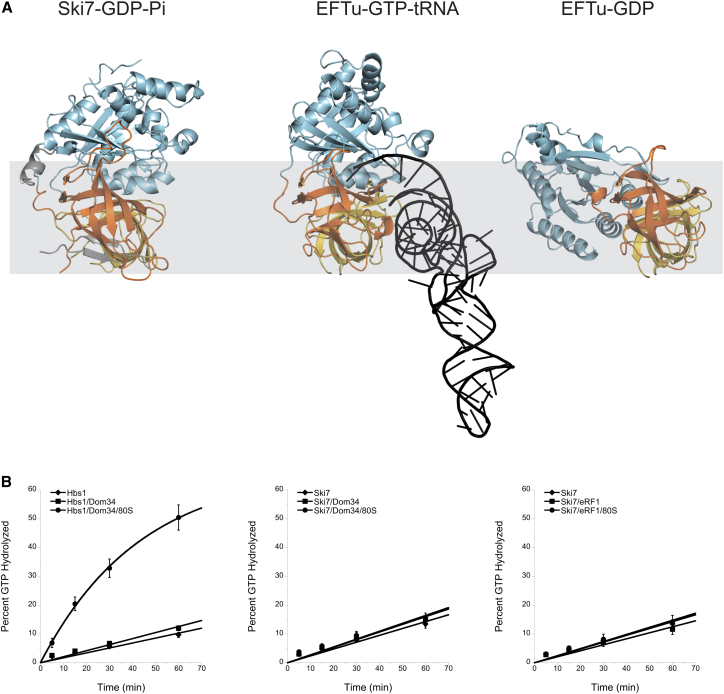
The Ski7_C_ Has the Domain Architecture of an Active trGTPase (A) The structures of Ski7_C_-GDP-P_i_, of EFTu-GDPPNP-tRNA (PDB: 1TTT) (Nissen et al., 1995) and of EFTu-GDP (PDB: 1TUI) (Polekhina et al., 1996) are shown after optimal superposition of domain II-III, in the same orientation and colors as in Figure 1B, right panel, RNA is shown in black. (B) GTPase activity of Ski7 and Hbs1 in the absence and presence of co-factors and 80S ribosomes. Ski7 GTPase stimulation was not observed by Dom34, eRF1, or 80S ribosomes (middle and right panel). Hbs1 GTPase activity and stimulation by Dom34 and ribosomes was included as a positive control (left panel). The Hbs1/Dom34/80S curve fits to a single exponential, while the other curves show linear fits. Error bars are ±1 SD from the mean across three experiments. (See also Figure S2).

We analyzed the domain orientation in the Ski7_C_ structures with respect to the conformations observed in canonical trGTPases. In particular, we compared Ski7_C_ with the crystal structures of *Thermus aquaticus* EFTu, which have been determined in an active state bound to GMPPNP and tRNA (Nissen et al., 1995) and in an inactive state bound to GDP (Polekhina et al., 1996). In the EFTu structures, the G domain undergoes a dramatic three-dimensional rigid-body motion relative to domains II and III (Figure 2, right and central panel). Structural rearrangements in the so-called switch regions in response to the presence or absence of the nucleotide γ-phosphate propagate with long-range effects, resulting in the reorganization of inter-domain contacts. In Ski7_C_, domain G and domains II-III adopt a very similar architecture to that observed in the active-state snapshot of EFTu (Figure 2, left and central panels). When comparing the additional interactions with extended segments, EFTu lacks the equivalent of the N-terminal helix α_0_ of Ski7_C_ but features a similar loop that protrudes from domain II and binds the G domain as well as the 3′ end of the tRNA. We conclude that the conformation of Ski7_C_ in both the GTP-bound and the GDP-P_i_-bound structures parallels the active conformation of a canonical trGTPase.

In the active conformation, canonical trGTPases bind their cofactors at a cleft between the G domain and the barrel domains. Ski7_C_ has a cleft at the corresponding position as the cofactor-binding surface of known trGTPases, but the detailed shape and electrostatic properties are distinct (Figure S2B). Thus, Ski7 is unlikely to use the same cofactors. Indeed, neither eRF1 nor Dom34 could augment the intrinsic GTPase activity of Ski7 in an experimental setup that robustly induces Hbs1 GTPase activity (Figure 2B).

### Ski7_C_ Can Bind Either Intact or Cleaved GTP

**Figure 3:**
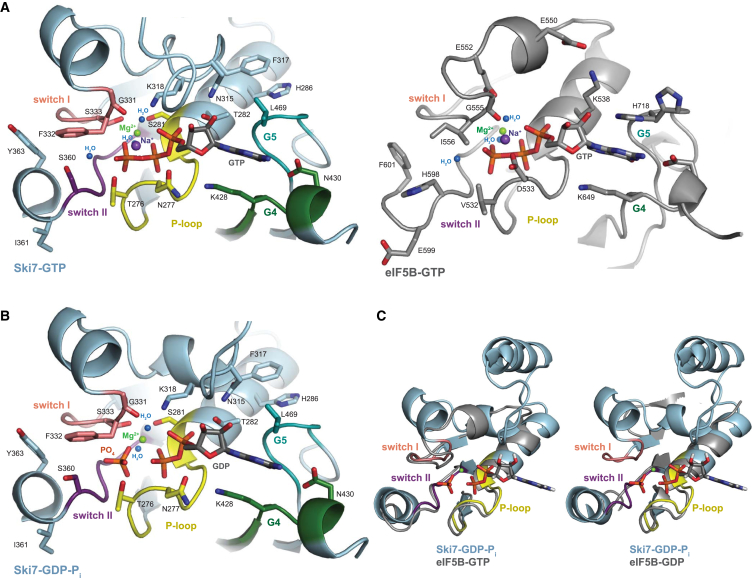
The Active Site of Ski7_C_ (A) Zoom-in view of the GTP-binding site in the Ski7_C_-GTP structure (left panel) compared with that of the trGTPase eIF5B in the active GTP-bound structure (right panel) (PDB: 4TMW) (Kuhle and Ficner, 2014b). The two molecules are shown in the same orientation after optimal superposition of their G domains. In Ski7 the five motifs of G domains are colored in yellow (P loop, also known as G1 motif), salmon (switch I, also known as G2 motif), violet (switch 2, also known as G3 motif), green (G4), and teal (G5). The Ski7 active site shows the presence of the divalent and monovalent cations and waters at the corresponding structural positions observed in eIF5B-GTP. (B) Zoom-in view of the GTP-binding site in the Ski7_C_-GDP-P_i_ structure (left panel) compared in the same orientation as the molecules above. (C) Zoom-in on the nucleotide-binding regions in the Ski7_C_-GDP-P_i_ structure (P loop, switch I and switch II), superposed with the corresponding regions of eIF5B in the active state (bound to GTP, PDB: 4TMW [Kuhle and Ficner, 2014b], left side) and in the inactive state (bound to GDP, PDB: 4NCL [Kuhle and Ficner, 2014a], right side). Proteins are in cartoon representation and the displayed nucleotide and ions are those bound by Ski7. The G motifs of Ski7_C_ are colored highlighting their similarity to the active conformation of eIF5B. (See also Figure S3).

Superposition of the Ski7_C_-GTP and Ski7_C_-GDP-P_i_ structures shows that the guanosine moieties and the α- and β-phosphates of the two nucleotides are identically placed in the nucleotide-binding site (Figures S2B and S2C). The γ-phosphate of the GTP-bound structure and the inorganic phosphate of the GDP-P_i_-bound structure are adjacent to each other, but they do not coincide. In Ski7_C_-GTP, the γ-phosphate is connected covalently to the β-phosphate (with the canonical phosphate-phosphate distance of 2.8 Å). In Ski7_C_-GDP-P_i_, the inorganic phosphate is clearly separated from the β-phosphate of the GDP (with a phosphate-phosphate distance of 4.2 Å) (Figures 3A, 3B, and S2A).

We compared the nucleotide-binding site in the G domain of Ski7 with that of eIF5B, a trGTPase involved in subunit joining and whose G domain structure has been characterized at high resolution in several apo and nucleotide-bound states (Kuhle and Ficner, 2014a, 2014b) (Figure 3A). G domains have five consensus sequence motifs (G1–G5) (Bourne et al., 1991). Motif G1 (also known as the P loop or Walker A motif) interacts with the α- and β-phosphates of the nucleotide. Motif G2 and motif G3 (also known as the Walker B motif) bind the γ-phosphate of GTP and correspond to the switch I and switch II regions. Motifs G4 and G5 interact with the guanine base. The polypeptide backbones of the P loop, G4, and G5 motifs have essentially the same conformations in all nucleotide-bound eIF5B and Ski7_C_ structures (Figures 3A and 3B). In the case of switch I and switch II, the polypeptide backbone in Ski7_C_-GTP and Ski7_C_-GDP-P_i_ is very similar to that observed in eIF5B-GTP and differs from eIF5B-GDP (Figure 3C). The analysis thus indicates that both Ski7_C_ structures resemble the conformation of active GTP-bound trGTPases. In the case of the Ski7_C_-GDP-P_i_ structure, the complex either mimics a putative post-hydrolysis state (e.g. before release of the P_i_ product and conversion to an inactive GDP-bound state) or engages the small molecules supplied at high concentrations with the crystallization buffer (i.e. GDP and inorganic phosphate) to mimic a GTP-bound state.

### The Chemical Features of the Ski7 Nucleotide-Binding Site

**Figure 4:**
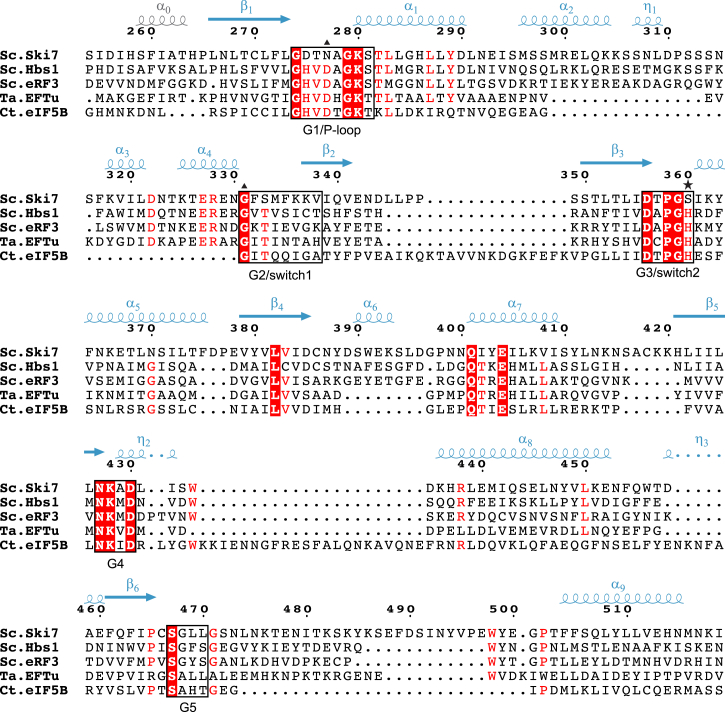
Structure-Based Sequence Alignment of the G Domain of Ski7_C_ and trGTPases The alignment includes *S. cerevisiae* (*Sc*) Ski7_C_ G domain (residues 254–518) and the corresponding regions of *Sc* Hbs1, *Sc* eRF3, *T. aquaticu*s (*Ta*) EFTu, and *Chaetomium thermophilum* (*Ct*) eIF5B. The secondary structure elements of Ski7_C_ are shown above the sequence, colored and labeled per domain. White letters on red background share 100% of identity within the shown sequences and red letters share 80% identity. The five conserved sequence motifs of G domains (G1–G5) are highlighted in black boxes. The star indicates the position of the catalytical histidine in the trGTPases; triangles indicate the position of the monovalent metal coordinating residues.

At the sequence level, the G motifs of Ski7 contain several unusual residues at positions that are highly conserved in Hbs1, eRF3, EFTu, and eIF5B (Figure 4). In most cases, residues that differ from the conserved amino acids of trGTPases are nevertheless engaged in similar interactions (Figure 3A, compare right and left panels). For example, a hydrophobic residue of the G5 motif (Leu469^Ski7^) packs against one side of the guanine base, at a position usually engaged in hydrophobic stacking interactions (Figure 3). In switch I, Ser333^Ski7^ coordinates the divalent cation and contributes to binding of the γ-phosphate, as does Gly359^Ski7^. In switch I, Phe332^Ski7^ maintains the chemical properties of the so-called hydrophobic gate of trGTPases (Berchtold et al., 1993; Kjeldgaard et al., 1993; Villa et al., 2009). Finally, in the P loop, Asn277^Ski7^ is at the position of Asp533^eIF5B^. In the eIF5B-GTP structure, Asp533^eIF5B^ and the main-chain carbonyl of Gly555^eIF5B^ (from switch I) coordinate a monovalent cation (either K^+^ or Na^+^) (Kuhle and Ficner, 2014b). The presence of a monovalent cation is thought to be a universal structural feature of active trGTPases (Kuhle and Ficner, 2014b), although it contributes little to catalysis both on and off the ribosome (Maracci et al., 2014; Åqvist and Kamerlin, 2015). In the Ski7-GTP structure, the electron density is consistent with the presence of a monovalent ion at the equivalent structural position, between Asn277^Ski7^ and the main-chain carbonyl of Gly331^Ski7^ (from switch I) (Figure 3A).

A distinguishing feature of Ski7 is the presence of Ser360^Ski7^ in switch II at the corresponding position of His598^eIF5B^. This active site histidine residue is conserved in trGTPases, and in the presence of the ribosome and an activating cofactor it re-orients its side chain to stabilize or position the catalytic water (Figures 3A and 4) (Berchtold et al., 1993; Daviter et al., 2003; Maracci et al., 2014; Villa et al., 2009). At the chemical level, it is conceivable that the function of the side chain of Ser360^Ski7^ might be to stabilize a putative catalytic water (Holliday et al., 2009) when re-oriented in the presence of the ribosome. Nevertheless, the shorter side chain would probably be suboptimal for catalysis. Another peculiarity of Ski7 is in the P loop, where Thr276^Ski7^ is at the position of Val532^eIF5B^ and engages the hydroxyl group in an unusual interaction with the nucleotide γ-phosphate (Figure 3A, left panel, and 3B). Such interaction might help to stabilize the GTP-bound state of Ski7. Analysis of Ski7_C_ by high-performance liquid chromatography indeed indicated that the protein co-purified from *Escherichia coli* with bound GTP while purified Hbs1 did not carry any nucleotide (Figure S3A). Addition of non-cleavable nucleotide analogs during alkaline phosphatase treatment and a subsequent thermostability assay (Thermofluor) showed a decrease in the melting temperature (T_m_) of GMPCP-bound Ski7C (44.0°C) in comparison with GMPPNP-bound protein (47.6°C), indicating a preference for the GTP-bound state (Figure S3B). Nucleotide-free protein showed an even lower T_m_ of 42.8°C and precipitated at higher concentrations.

### Conclusions

trGTPases have characteristic features. At the biochemical level, they have low hydrolysis activity in isolation and require the ribosome and a specific cofactor for robust activity. At the structural level, they have a characteristic domain composition, a characteristic conformation in the active GTP-bound state, and a characteristic active site with a monovalent cation in addition to the universal divalent cation present in other GTPases. The work we report here indicates that the C-terminal G domain of *S. cerevisiae* Ski7 possesses the structural features of a canonical trGTPase not only in terms of domain structure, as expected from sequence analysis, but also in terms of conformation. Both the GTP-bound and the GDP-P_i_-bound complexes of Ski7_C_ adopt the conformation typical of trGTPases in the active GTP-bound state and show an overall similar nucleotide-binding pocket. The major differences are the presence of a different polar residue at the putative catalytic center (a serine instead of a histidine) and an additional polar residue near the γ-phosphate of GTP (a threonine instead of a valine). We envision two possible scenarios of how Ski7_C_ might function. In one scenario, Ski7 could be a trGTPase-like protein in the full sense despite the possession of non-canonical active site residues. In this case, it would be capable of GTP hydrolysis and of switching between active and inactive conformations in the context of the ribosome and of an appropriate cofactor. Such a cofactor would be expected to function analogously to Dom34 and eRF1 but in addition would likely have to stabilize the GDP-bound form of the protein. Alternatively, in a second scenario, Ski7 could have evolved as a GTP-binding protein, i.e. as a pseudo-trGTPase. Here, a Ski7 cofactor would be expected to regulate the interaction with the ribosome with an altogether different mechanism, which does not rely on a conformational switch of the Ski7_C_ domains. Finally, Ski7 might even function without any cofactor. Although the biochemical data available so far would favor the concept of Ski7 as a stable GTP binder, only the identification of the cofactors that might regulate the function of the Ski7_C_ in NSD will allow for clarification of the mechanisms.

## Experimental Procedures

### Protein Expression and Purification

Ski7_C_ (254–747) was expressed as a fusion protein with an N-terminal thioredoxin polypeptide cleavable with Prescission Protease. The expression was carried out in *E. coli* BL21 Gold pLyS cells (Stratagene) using Terrific Broth and inducing with 0.1 mM isopropyl β-D-1-thiogalactopyranoside at 18°C for 16 hr. Expression of selenomethionine-derivatized protein was carried out in minimal medium upon addition of amino acids and 50 mg/l selenomethionine prior to induction. Ski7_C_ was purified using a Nickel affinity step (His FF, GE Healthcare) followed by cleavage of the tag with Prescission Protease, purification on heparin resin (Heparin HP, GE Healthcare) and a final size exclusion chromatography step (Superdex S200, GE Healthcare) using 20 mM HEPES (pH 7.5), 100 mM NaCl.

### Crystallization and Structure Determination

Ski7_C_ was crystallized at a concentration of 7 mg/ml with 5 mM nucleotide (GDP or GTP), 1 mM MgCl_2_, and 5 mM Tris(2-carboxyethyl)phosphine. Crystals grew in 100 mM HEPES (pH 7.0), 700 mM NaH_2_PO_4_/K_2_HPO_4_, and 3% ethylene glycol. The crystals were cryo-protected in the presence of 25% ethylene glycol and flash-cooled in liquid nitrogen. In the case of the manganese-containing crystals, 10 mM MnCl_2_ was included in the cryo-protectant. All X-ray diffraction data were collected at 100 K at the Swiss Light Source (SLS) synchrotron in Villigen, Switzerland. The single anomalous diffraction experiment was performed by collecting a dataset at the peak wavelength of the selenium K edge and another dataset was collected from the same crystal at the manganese K edge. The data were processed and scaled with XDS (Kabsch, 2010). The crystals belong to the space group C222_1_ containing one molecule in the asymmetric unit. The data processing statistics are summarized in Table 1. The structure was determined and refined with standard crystallographic packages (detailed in the Supplemental Methods).

## Author Contributions

The structure was determined and analyzed by E.K. Activity assays were prepared by A.S. Experiments were performed under the direction of E.C and R.G. E.K and E.C wrote the article.

## Figures and Tables

**Table 1:** X-Ray Data Collection and Refinement Statistics

Dataset	Ski7C-GTP	Ski7-GDP-P_i_
Wavelength (Å)	1.000	1.000
Resolution range (Å)	45.58–2.251 (2.331–2.251)	73.72–2.181 (2.259–2.181)
Space group	C 2 2 21	C 2 2 21
a, b, c (Å)	91.150, 123.106, 104.967	93.136, 120.617, 105.269
α, β, γ (°)	90, 90, 90	90, 90, 90
Total reflections	188,256 (18,033)	205,103 (18,270)
Unique reflections	27,933 (2,656)	31,136 (2,983)
Multiplicity	6.7 (6.8)	6.6 (6.1)
Completeness (%)	98.47 (94.89)	99.71 (97.48)
Mean I/sigma (I)	13.36 (1.25)	15.98 (0.86)
CC1/2	0.998 (0.487)	0.999 (0.517)

**Refinement**		

*R*_work_ (%)	0.2047	0.2172
*R*_free_ (%)	0.2387	0.2460
Protein residues	254–476, 490–636, 645–747	255–476, 492–636, 645–747
Ligands	GTP, Mg^2+^, Na^+^, triethylene glycol	GDP, P_i_, Mg^2+^
Water	96	10

**Stereochemistry**		

RMS (bonds)	0.003	0.003
RMS (angles)	0.76	0.70
Ramachandran favored (%)	98	97
Ramachandran outliers (%)	0	0

Values for the highest-resolution shell are given in parentheses.

## Accession Numbers

The coordinates and structure factors of Ski7_C_-GDP-P_i_ and Ski7_C_-GTP have been deposited in the PDB with accession codes PDB: 4ZKD and 4ZKE, respectively.

## Supplemental Information

Document S1. Figures S1–S3 and Supplemental Methods

Document S2. Article plus Supplemental Information
