# Supplementary material for: Saccharomyces cerevisiae Ski7 Is a GTP-Binding Protein Adopting the Characteristic Conformation of Active Translational GTPases
Source: Structure. 2015 Jul 7;23(7):1336–43. doi: 10.1016/j.str.2015.04.018 (PMC4509514; doi:10.1016/j.str.2015.04.018)
Supplement: Document S1. Figures S1–S3 and Supplemental Methods [file mmc1.pdf]

Structure, Volume *23*

Supplemental Information

Saccharomyces cerevisiae Ski7 Is a GTP-Binding Protein Adopting the Characteristic Conformation of Active Translational GTPases

Eva Kowalinski, Anthony Schuller, Rachel Green, and Elena Conti

SUPPLEMENTAL DATA

SUPPLEMENTAL FIGURES

FIGURE S1

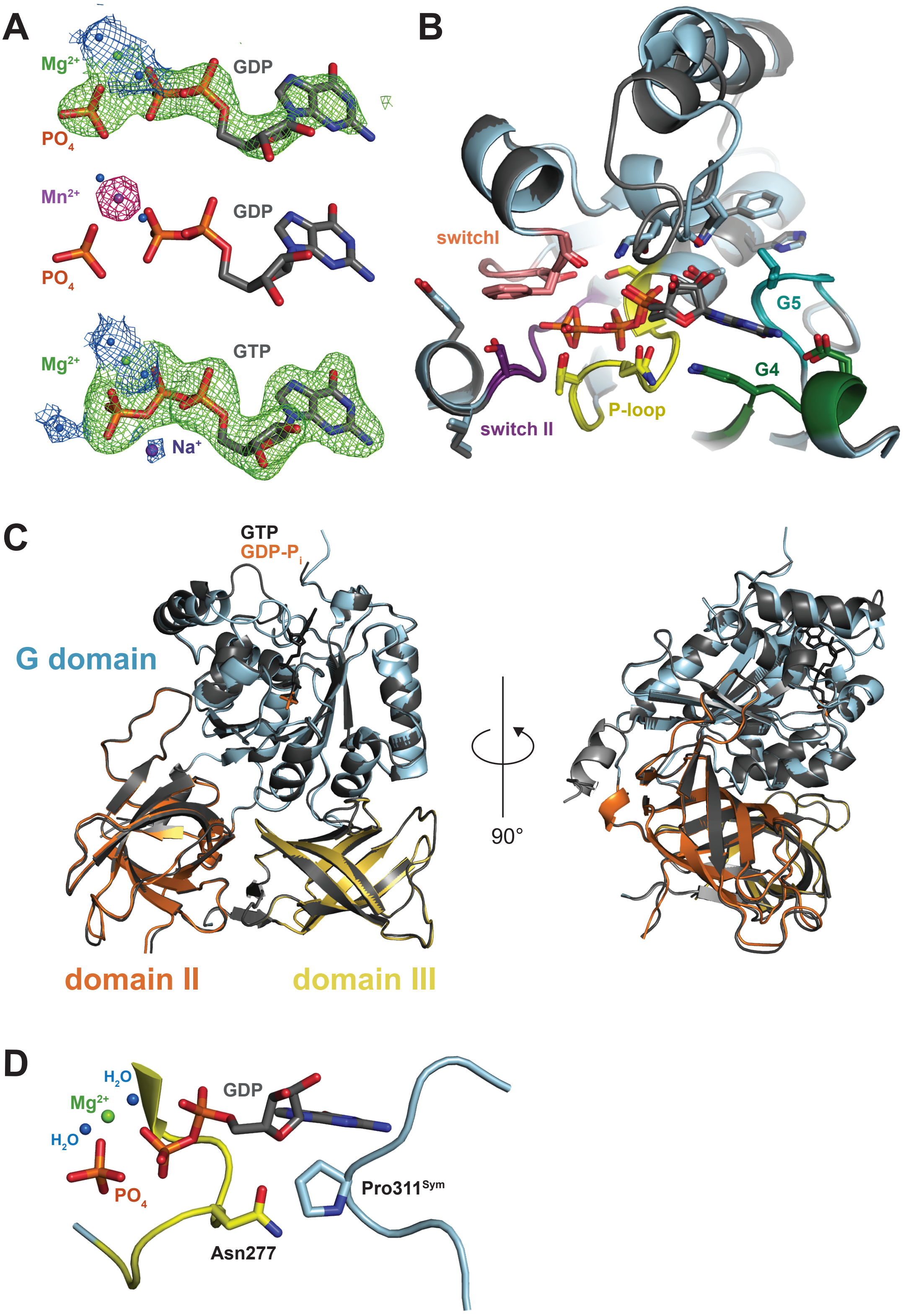

FIGURE S2

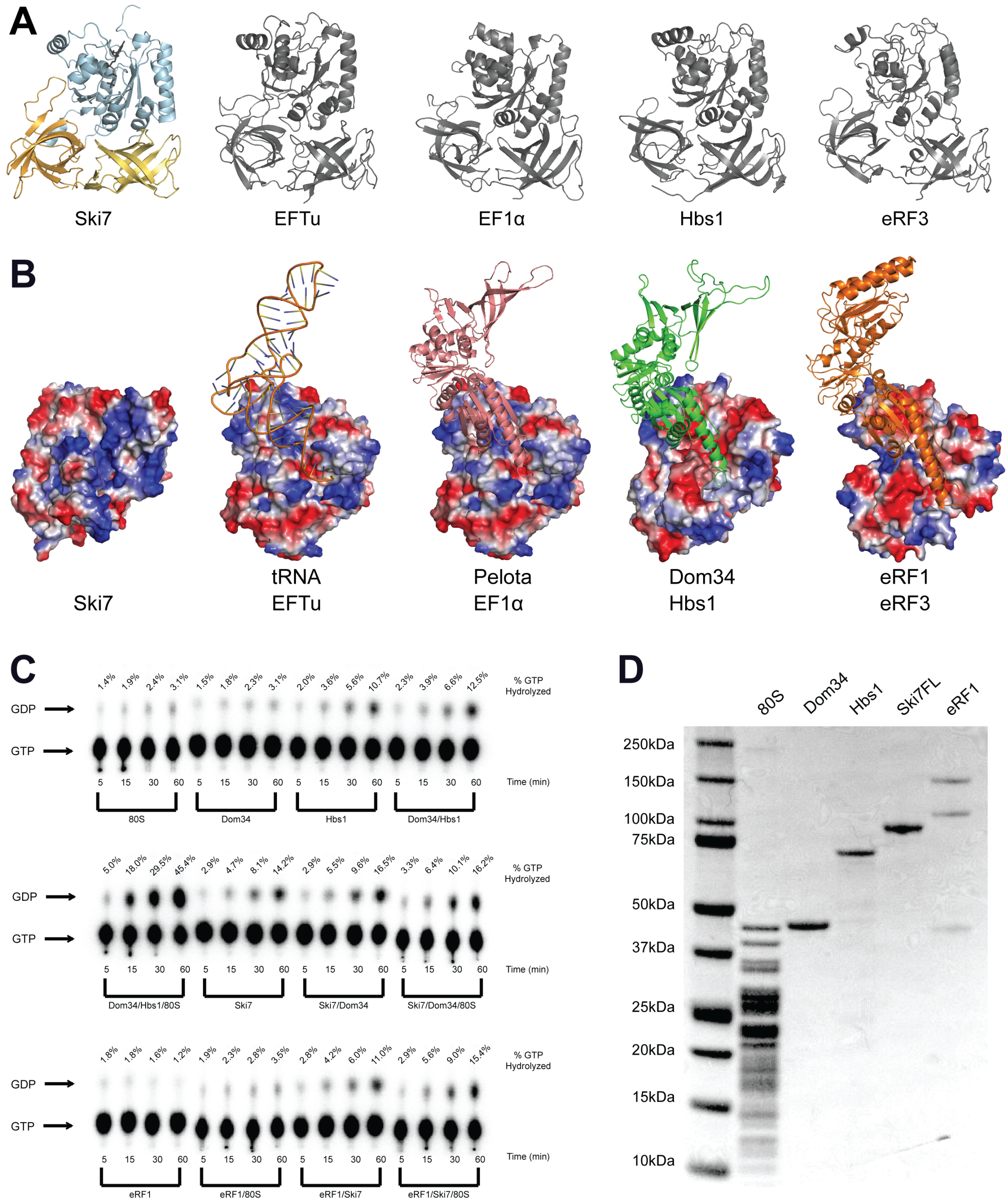
A
Ski7
EFTu
EF1α
Hbs1
eRF3
B
Ski7
tRNA
EFTu
Pelota
EF1α
Dom34
Hbs1
eRF1
eRF3
C
1.4% 1.9% 2.4% 3.1% 1.5% 1.8% 2.3% 3.1% 2.0% 3.6% 5.6% 10.7% 2.3% 3.9% 6.6% 12.5%
% GTP Hydrolyzed
GDP
GTP
5 15 30 60 5 15 30 60 5 15 30 60 5 15 30 60
Time (min)
80S
Dom34
Hbs1
Dom34/Hbs1
5.0% 18.0% 29.5% 45.4% 2.9% 4.7% 8.1% 14.2% 2.9% 5.5% 9.6% 16.5% 3.3% 6.4% 10.1% 16.2%
% GTP Hydrolyzed
GDP
GTP
5 15 30 60 5 15 30 60 5 15 30 60 5 15 30 60
Time (min)
Dom34/Hbs1/80S
Ski7
Ski7/Dom34
Ski7/Dom34/80S
1.8% 1.8% 1.6% 1.2% 1.9% 2.3% 2.8% 3.5% 2.8% 4.2% 6.0% 11.0% 2.9% 5.6% 9.0% 15.4%
% GTP Hydrolyzed
GDP
GTP
5 15 30 60 5 15 30 60 5 15 30 60 5 15 30 60
Time (min)
eRF1
eRF1/80S
eRF1/Ski7
eRF1/Ski7/80S
D
80S
Dom34
Hbs1
Ski7FL
eRF1
250kDa
150kDa
100kDa
75kDa
50kDa
37kDa
25kDa
20kDa
15kDa
10kDa

FIGURE S3

A

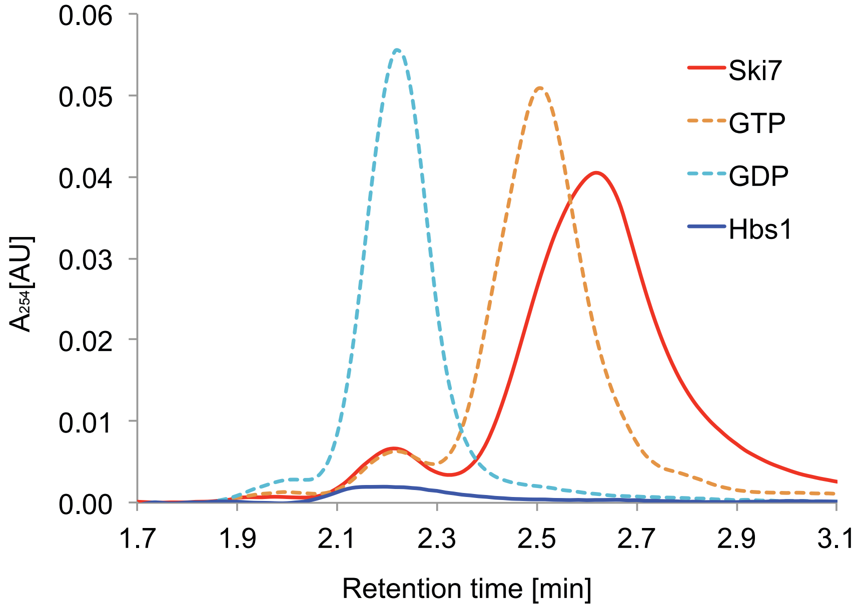
0.06
0.05
0.04
0.03
0.02
0.01
0.00
A254[AU]
1.7
1.9
2.1
2.3
2.5
2.7
2.9
3.1
Retention time [min]
Ski7
GTP
GDP
Hbs1

B

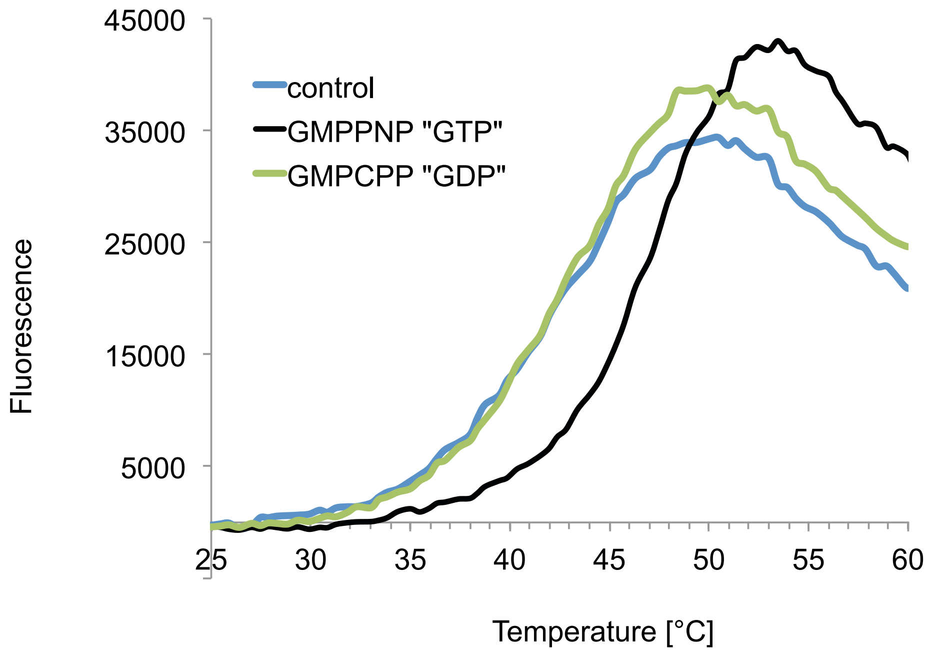
45000
35000
25000
15000
5000
Fluorescence
25
30
35
40
45
50
55
60
Temperature [°C]
control
GMPPNP "GTP"
GMPCPP "GDP"

SUPPLEMENTAL FIGURE LEGENDS

FIGURE S1 (related to Figure 1)

Detailed structural analysis of Ski7 bound to GTP and to GDP-P_i

(A) Electron density of the ligands. Upper and middle panel: $Ski7_C$-GDP-P_i, lower $Ski7_C$-GTP. GDP and P_i are shown as sticks, Mg^{2+} (green), H_2O (blue), Na^+ (purple), Mn^{2+} (purple) as spheres. 2Fo-Fc map at $\sigma = 0.8$, displayed in a radius of 1.6 Å around the Na^+ and Mg^{2+} coordinating waters in blue; Fo-Fc map at $\sigma = 2.5$ after carrying out the last refinement step omitting the nucleotide ligand and Na^+, displayed in a radius of 1.6 Å around the nucleotide, inorganic phosphate and Na^+ in green; lower panel: anomalous difference map for Manganese of a $Ski7_C$-GDP-P_i crystal that has been soaked with $MnCl_2$ prior to cryo-cooling at $\sigma = 15.0$, displayed in a radius of 1.6 Å around the Manganese ion in pink.

(B) Zoom-in view of the GTP-binding site of the superposed GTP and GDP-P_i structures. $Ski7_C$-GTP in cyan and $Ski7_C$-GDP-P_i in grey. Other colors and residues as in main Figure 4.

(C) Superposition of cartoon representations of $Ski7_C$-GTP colors as in Figure 1 and $Ski7_C$-GDP-P_i in gray shown in two orientations, related by a 90° anti-clockwise rotation around a vertical axis. GTP is shown in stick representation in black and GDP-P_i in orange.

(D) Crystal contact between Asn277 and Pro311 of a symmetry related $Ski7_C$-GDP-P_i molecule. GDP, P_i, Asn277 and Pro311are shown as sticks, Mg^{2+} (green) and H_2O (blue) as spheres.

FIGURE S2 (related to Figure 2)

The G domain and domains II-III of Ski7$_C$ are similar to those in active trGTPases, but a co-factor could not be identified

(A) The following GTPase structures were optimally aligned based on domains II and III onto the Ski7$_C$ structure and their cartoon representation is shown omitting their co-factors: EFTu (pdb code 1TTT, in complex with tRNA (Nissen et al., 1995)), EF1α (pdb code 3WXM, in complex with Pelota (Kobayashi et al., 2010)), Hbs1 (pdb code 3MCA, in complex with Dom34 (Chen et al., 2010)) and eRF3 (pdb code 4CRN, in complex with eRF1 (Preis et al., 2014)).

(B) The surface potential of different trGTPases was calculated in pymol (DeLano) and compared to Ski7 for possible co-factor binding. See Supplemental Figure 2 for the corresponding pdb accession codes.

(C) Raw data of Figure 2B. GTP hydrolysis assays with Ski7 and potential cofactors were performed using 1 μM ribosomes, 2 μM Dom34, 3 μM Hbs1, 2 μM Ski7$_{fl}$, and 2 μM eRF1 in various combinations with 1x Buffer E (20 mM Tris-HCl, pH 7.5, 100 mM KOAc pH 7.6, 2.5 mM $Mg(OAc)_2$, 2 mM DTT, 0.25 mM spermidine), 1 mM GTP, and 5 nM ^{32}P-αGTP. Reactions were quenched after 5 min, 15 min, 30 min, and 60 min with 30% formic acid. Samples were spotted on PEI-Cellulose F TLC plates (EMD Millipore) and analyzed in 0.5 M KH_2PO_4 pH 3.5. TLC plates were developed using a Typhoon FLA 9500 phosphorimaging system and quantified using ImageQuant TL (GE Healthcare Life Sciences). For the assay Ski7$_{FL}$ (1-747) was expressed as N-terminal Thioredoxin-tagged and C-terminal Strep-tagged protein using a pET-derived expression vector in E. coli BL21 Gold pLyS cells (Stratagene). Percent GTP hydrolysis is indicated above each lane.

(D) Coomassie stained gel of the proteins used in the TLC assay.

FIGURE S3 (related to Figure 3)

Nucleotide binding properties of Ski7

(A) High pressure liquid chromatography (HPLC) elution profiles with controls. Nucleotide load of $Ski7_C$ and GST-Hbs1 was analyzed at 254 absorption nm by HPLC on a RP-18-HPLC column in a buffer containing 7.5% acetonitrile, 10 mM tetrabutylammonium bromide and 100 mM KP_i buffer (pH 6.5) (Ahmadian et al., 1999). Amounts of 2 nmol nucleotides, 2 nmols $Ski7_C$ and 1.7 nmols of GST-Hbs1 were loaded.

(B) Melting curves of $Ski7_C$ after nucleotide exchange. For nucleotide exchange, 2 mg/ml of $Ski7_C$ were incubated for 2.5 h at room temperature with rSAP (NEB, 1U per 1mg of of $Ski7_C$) under addition of either no nucleotide, GMPCPP (to mimic GDP) or GMPPNP (to mimic GTP). Subsequently, 5 µl of these reactions, 35x of Sypro Orange (Invitrogen) and 45 µL of size exclusion chromatography buffer were mixed and sealed in a 96-well PCR plate (Eppendorf). In a real-time PCR system (Eppendorf) reactions were heated from 20 °C to 80 °C in increments of 0.2 °C. Fluorescence changes were monitored simultaneously. The wavelengths for excitation and emission were 470 and 550 nm, respectively. The temperature midpoint for the protein unfolding transition (Tm) was read out using the minimum of the derivative of the corresponding curve (Ericsson et al., 2006).

SUPPLEMENTAL METHODS

The program autoSHARP/SHARP was used to find 6 Selenium atom sites and to calculate phases to 3.5 Å (Vonrhein et al., 2007). An initial model was generated with Phenix Autosol (Adams et al., 2010) and the resulting electron density map was used

for automated model building in BUCCANEER (Cowtan, 2006) and manual model building with COOT (Emsley and Cowtan, 2004). The refinement was performed with Phenix Refine (Afonine et al., 2012) and the stereochemical quality was assessed by MolProbity (Davis et al., 2007). The refined coordinates of $Ski7_C$ from the GDP-P_i–bound structure were refined against the GTP-bound data, and manual building with COOT (Emsley and Cowtan, 2004) allowed modeling of the nucleotide ligand and metals (the occupancy of the Na+ ion was refined to 0.64). In the Ski7-GDP-Pi structure, no density at the corresponding position of the monovalent cation was detected, likely due to the conformation of $Asn277^{Ski7}$, whose side chain is rotated to contact a symmetry-related molecule (Figure S1D).

SUPPLEMENTAL REFERENCES

Adams, P.D., Afonine, P.V., Bunkóczi, G., Chen, V.B., Davis, I.W., Echols, N., Headd, J.J., Hung, L.W., Kapral, G.J., Grosse-Kunstleve, R.W., et al. (2010). PHENIX: a comprehensive Python-based system for macromolecular structure solution. Acta Crystallogr. D Biol. Crystallogr.

Afonine, P.V., Grosse-Kunstleve, R.W., Echols, N., Headd, J.J., Moriarty, N.W., Mustyakimov, M., Terwilliger, T.C., Urzhumtsev, A., Zwart, P.H., and Adams, P.D. (2012). Towards automated crystallographic structure refinement with phenix.refine. Acta Crystallogr. D Biol. Crystallogr. *68*, 352–367.

Ahmadian, M.R., Zor, T., Vogt, D., Kabsch, W., Selinger, Z., Wittinghofer, A., and Scheffzek, K. (1999). Guanosine triphosphatase stimulation of oncogenic Ras mutants. Proc. Natl. Acad. Sci. U.S.a. *96*, 7065–7070.

Cowtan, K. (2006). The Buccaneer software for automated model building. 1. Tracing protein chains. Acta Crystallogr. D Biol. Crystallogr. *62*, 1002–1011.

Davis, I.W., Leaver-Fay, A., Chen, V.B., Block, J.N., Kapral, G.J., Wang, X., Murray, L.W., Arendall, W.B., Snoeyink, J., Richardson, J.S., et al. (2007). MolProbity: all-atom contacts and structure validation for proteins and nucleic acids. Nucleic Acids Res. *35*, W375–W383.

DeLano, W.L. The PyMOL Molecular Graphics System; DeLano Scientific LLC: Palo Alto, CA, 2008.

Emsley, P., and Cowtan, K. (2004). Coot: model-building tools for molecular graphics. Acta Crystallogr. D Biol. Crystallogr. *60*, 2126–2132.

Ericsson, U.B., Hallberg, B.M., Detitta, G.T., Dekker, N., and Nordlund, P. (2006). Thermofluor-based high-throughput stability optimization of proteins for structural studies. Anal. Biochem. *357*, 289–298.

Vonrhein, C., Blanc, E., Roversi, P., and Bricogne, G. (2007). Automated structure solution with autoSHARP. Methods Mol. Biol. *364*, 215–230.
