# Supplementary material for: Saccharomyces cerevisiae Ski7 Is a GTP-Binding Protein Adopting the Characteristic Conformation of Active Translational GTPases
Source: Structure. 2015 Jul 7;23(7):1336–43. doi: 10.1016/j.str.2015.04.018 (PMC4509514; doi:10.1016/j.str.2015.04.018)
Supplement: Document S2. Article plus Supplemental Information [file mmc2.pdf]

Short Article

Structure

Saccharomyces cerevisiae Ski7 Is a GTP-Binding Protein Adopting the Characteristic Conformation of Active Translational GTPases

Graphical Abstract

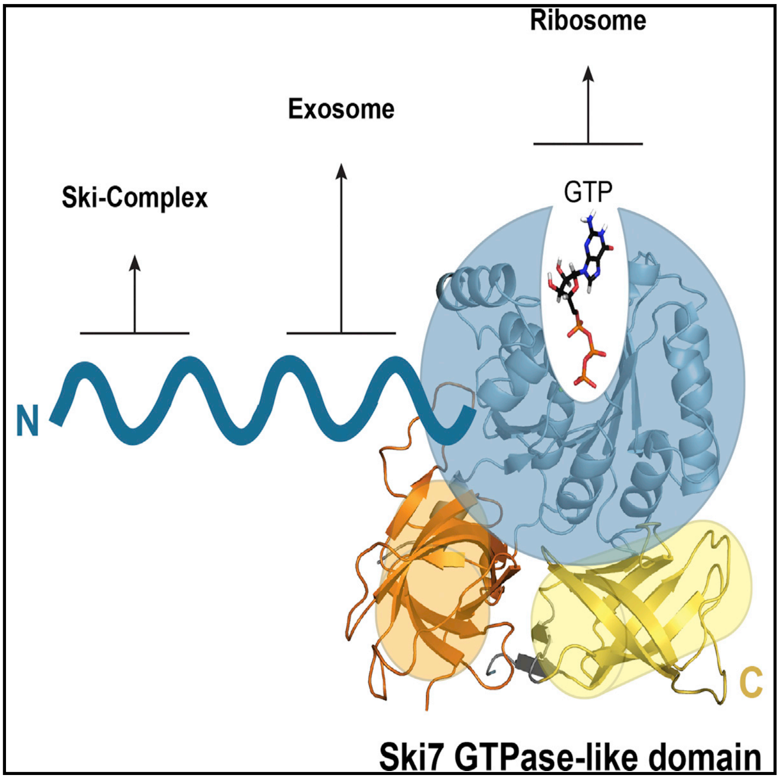

Authors

Eva Kowalinski, Anthony Schuller, Rachel Green, Elena Conti

Correspondence

conti@biochem.mpg.de

In Brief

Kowalinski et al. report the crystal structure of the GTPase-like domain of yeast Ski7, a protein module involved in non-stop decay, a ribosome-dependent mRNA surveillance pathway. This study shows that Ski7 interacts stably with guanosine triphosphate (GTP) and adopts the overall structural features of ribosome-associated GTPases.

Highlights

- The Ski7 C-terminal region adopts the conformation of active translational GTPases
- Ski7 features the monovalent cation characteristic of active translational GTPases
- Ski7 has unusual features in the nucleotide-binding site and binds GTP stably
- Ski7 might have evolved as a pseudo-trGTPase with enhanced GTP-binding properties

Accession Numbers

4ZKD
4ZKE

Kowalinski et al., 2015, Structure *23*, 1336–1343
July 7, 2015 ©2015 The Authors
http://dx.doi.org/10.1016/j.str.2015.04.018

Saccharomyces cerevisiae Ski7 Is a GTP-Binding Protein Adopting the Characteristic Conformation of Active Translational GTPases

Eva Kowalinski,[1] Anthony Schuller,[2] Rachel Green,[2] and Elena Conti[1,*]
[1]Department of Structural Cell Biology Department, Max Planck Institute of Biochemistry, Am Klopferspitz 18, 82152 Martinsried, Germany
[2]Howard Hughes Medical Institute, Department of Molecular Biology and Genetics, Johns Hopkins University School of Medicine, Baltimore, MD 21205, USA
*Correspondence: conti@biochem.mpg.de
http://dx.doi.org/10.1016/j.str.2015.04.018
This is an open access article under the CC BY license (http://creativecommons.org/licenses/by/4.0/).

SUMMARY

Ski7 is a cofactor of the cytoplasmic exosome in budding yeast, functioning in both mRNA turnover and non-stop decay (NSD), a surveillance pathway that degrades faulty mRNAs lacking a stop codon. The C-terminal region of Ski7 (Ski7$_C$) shares overall sequence similarity with the translational GTPase (trGTPase) Hbs1, but whether Ski7 has retained the properties of a trGTPase is unclear. Here, we report the high-resolution structures of Ski7$_C$ bound to either intact guanosine triphosphate (GTP) or guanosine diphosphate-P$_i$. The individual domains of Ski7$_C$ adopt the conformation characteristic of active trGTPases. Furthermore, the nucleotide-binding site of Ski7$_C$ shares similar features compared with active trGTPases, notably the presence of a characteristic monovalent cation. However, a suboptimal polar residue at the putative catalytic site and an unusual polar residue that interacts with the γ-phosphate of GTP distinguish Ski7 from other trGTPases, suggesting it might function rather as a GTP-binding protein than as a GTP-hydrolyzing enzyme.

INTRODUCTION

The decay of cytoplasmic mRNAs regulates the output of eukaryotic gene expression in terms of both quantity and quality. In general, mRNA turnover modulates the abundance of normal transcripts in the cell and thereby the quantity of the proteins they encode (reviewed in Garneau et al., 2007; Houseley and Tollervey, 2009; Parker, 2012). In addition, eukaryotic cells have evolved quality-control mechanisms that prompt the decay of defective mRNAs. These surveillance pathways act at translating ribosomes and recognize different types of defects, for example the absence of a stop codon (non-stop decay [NSD]), the presence of a premature stop codon (nonsense-mediated decay [NMD]) or the presence of features that cause abnormal stalling of the translation machinery (no-go decay [NGD]) (reviewed in Inada, 2013; Kervestin and Jacobson, 2012; Klauer and van Hoof, 2012; Lykke-Andersen and Bennett, 2014; Popp and Maquat, 2013; Schweingruber et al., 2013; Shoemaker and Green, 2012). In both normal and aberrant situations, enzymatic machineries eventually degrade the body of the mRNA either from the 5′ end (via Xrn1) or from the 3′ end (via the exosome) (reviewed in Garneau et al., 2007; Houseley and Tollervey, 2009).

The exosome core complex is a ten-subunit assembly endowed with processive 3′–5′ exoribonuclease activity (Mitchell et al., 1997; reviewed in Januszyk and Lima, 2014; Makino et al., 2013). In the cytoplasm, the exosome functions together with the Ski complex, a 4-subunit protein complex centered around an RNA helicase (Brown et al., 2000; Halbach et al., 2013). In *Saccharomyces cerevisiae*, the interaction between the exosome and the Ski complex is mediated by Ski7 (Araki et al., 2001; van Hoof et al., 2000). While the exosome and the Ski complexes are evolutionary conserved, Ski7 has long been thought to be unique to *Saccharomyces* species. Recently, however, it has been shown that *Lachancea kluyveri* expresses a Ski7 protein by alternative splicing of the *HBS1* gene (Atkinson et al., 2008; Marshall et al., 2013), raising the possibility that Ski7 might have a wider phylogenetic distribution than currently thought.

Ski7 is a multidomain protein. The N-terminal portion contains the regions that mediate binding to the exosome and Ski complexes (Araki et al., 2001) and is required for all exosome-mediated RNA decay pathways, including general mRNA turnover (van Hoof et al., 2002). The C-terminal part contains a GTPase domain and is required specifically in the NSD pathway (Frischmeyer et al., 2002; van Hoof et al., 2000). Interestingly in this context, the closest paralog of Ski7 is Hbs1, a translational GTPase (trGTPase) also involved in aberrant translation termination (reviewed in Hoshino, 2012). Hbs1 was originally implicated in NGD (Doma and Parker, 2006) and has recently been shown to participate in both NSD (Saito et al., 2013; Tsuboi et al., 2012) and the rescue of ribosomes arrested at the 3′ end of truncated mRNAs or stalled in the 3′ UTR (Guydosh and Green, 2014; Shoemaker et al., 2010).

Like all known GTPases, Hbs1 switches between an active GTP-bound conformation and an inactive guanosine diphosphate (GDP)-bound conformation (reviewed in Wittinghofer and Vetter, 2011). On its own, Hbs1 has negligible intrinsic GTPase activity (Shoemaker et al., 2010). However, the GTP hydrolysis reaction of Hbs1 is greatly stimulated by the presence of Dom34 and the ribosome, which together fulfill the function of a composite GTPase-activating protein (Shoemaker et al., 2010). In this sense, the Hbs1-Dom34 complex is analogous to

Table 1. X-Ray Data Collection and Refinement Statistics

Dataset	Ski7C-GTP	Ski7-GDP-P_i
Wavelength (Å)	1.000	1.000
Resolution range (Å)	45.58–2.251 (2.331–2.251)	73.72–2.181 (2.259–2.181)
Space group	C 2 2 21	C 2 2 21
a, b, c (Å)	91.150, 123.106, 104.967	93.136, 120.617, 105.269
α, β, γ (°)	90, 90, 90	90, 90, 90
Total reflections	188,256 (18,033)	205,103 (18,270)
Unique reflections	27,933 (2,656)	31,136 (2,983)
Multiplicity	6.7 (6.8)	6.6 (6.1)
Completeness (%)	98.47 (94.89)	99.71 (97.48)
Mean I/sigma (I)	13.36 (1.25)	15.98 (0.86)
CC1/2	0.998 (0.487)	0.999 (0.517)
Refinement		
R_{work} (%)	0.2047	0.2172
R_{free} (%)	0.2387	0.2460
Protein residues	254–476, 490–636, 645–747	255–476, 492–636, 645–747
Ligands	GTP, Mg^{2+}, Na^{+}, triethylene glycol	GDP, P_i, Mg^{2+}
Water	96	10
Stereochemistry		
RMS (bonds)	0.003	0.003
RMS (angles)	0.76	0.70
Ramachandran favored (%)	98	97
Ramachandran outliers (%)	0	0

Values for the highest-resolution shell are given in parentheses.

the eRF1-eRF3 complex, which functions in translation termination at stop codons (reviewed in Zhou et al., 2012), and to the EFTu-aminoacyl-tRNA complex, which functions in translation elongation (reviewed in Voorhees and Ramakrishnan, 2013; Rodnina, 2009).

The overall similarities between Ski7 and its paralog Hbs1 are compelling. However, whether Ski7 is an active trGTPase is not clear, particularly because residues that are invariant in the catalytic site of canonical trGTPases are not conserved in Ski7. Furthermore, there is no cofactor known to associate with Ski7 that could fulfill a similar function to Dom34 or eRF1. In this work, we used structural approaches to shed light on the function of Ski7 and found that Ski7 can bind GTP and adopt the conformation of active GTP-bound trGTPases.

RESULTS AND DISCUSSION

Structure Determination of the C-Terminal GTPase-Like Region of Ski7

We engineered a C-terminal fragment of *S. cerevisiae* Ski7 that encompasses the predicted GTPase-like region (residues 254–747, thereby referred to as $Ski7_C$). $Ski7_C$ was incubated with either GTP or GDP for crystallization trials. We obtained crystals of $Ski7_C$ in the presence of GDP and magnesium ions using inorganic phosphate as precipitating agent. We solved the structure with a selenomethionine-based single-wavelength anomalous dispersion experiment and refined it at 2.2-Å resolution to R_{free} of 24.6%, R_{work} of 21.7%, and good stereochemistry (Table 1). The refined structure (referred to as $Ski7_C$-GDP-P_i) includes most of the $Ski7_C$ polypeptide chain (with the exception of short disordered loop regions) and also includes GDP, a molecule of inorganic phosphate (P_i) and an Mg^{2+} ion. Manganese was soaked into the crystal prior to data collection to substitute Mg^{2+} and to unambiguously identify the position of the divalent cation in the electron density using anomalous scattering (Figure S1A).

Using the same crystallization conditions, we also obtained crystals of $Ski7_C$ in the presence of GTP. The structure of $Ski7_C$-GTP was determined using the atomic coordinates of the protein chain from the $Ski7_C$-GDP-P_i structure. The refinement showed the presence of well-defined electron density for GTP and an Mg^{2+} ion, and weaker density, which was interpreted as a monovalent metal ion (Figures 1B and S2A). The final model was refined to 2.3-Å resolution with R_{free} of 23.9%, R_{work} of 20.5%, and good stereochemistry (Table 1). Except for a loop approaching the sugar moiety of the nucleotide that is involved in a crystal contact in the $Ski7_C$-GDP-P_i crystal (Figures S1B and S1D), the structure of $Ski7_C$ is essentially identical in the $Ski7_C$-GDP-P_i and $Ski7_C$-GTP complexes, superposing with a root-mean-square deviation of 0.53 Å over all atoms (Figure S1C). The description of $Ski7_C$ below thus refers to both structures, unless otherwise specified.

The $Ski7_C$ Structure Reveals a Domain Arrangement Typical of Active trGTPases

$Ski7_C$ is organized in three domains (Figure 1A). Domain I (residues 264–518) adopts the $\alpha\beta$ fold of GTP-binding domains (G domains), with a central six-stranded β sheet surrounded by α helices (Figure 1B). Domain II (residues 526–636) and domain III (residues 645–747) each adopt the structure of a closed β barrel (Figure 1B). The β barrels of $Ski7_C$ are positioned side by side, with two antiparallel β strands (β_A and β_B) wedged in between. The β_A and β_B strands are not adjacent in the sequence but form a small β sheet that interacts on one side with the barrel of domain II and on the other side with the barrel of domain III (Figure 1B). As observed for other GTPases of this family, domains II and III appear to form a single unit (Berchtold et al., 1993; Kjeldgaard et al., 1993).

Domains II and III both interact with the G domain (Figure 1B). The three domains interact intra-molecularly not only via their globular folds but also via extended segments. First, an α helix at the N terminus of the G domain (residues 254–264, α_0) packs against domain II and against the linker that connects the two domains. Second, a loop connecting strands β_4 and β_5 of domain II interacts with the side of the G domain. The trGTPases Hbs1, eRF3, EFTu, and eIF5B share a similar sequel of globular domains (Figure S2A). Several crystal structures of trGTPases have been determined in complex with different GTP analogs and with cofactors (Figure S2B) (Chen et al., 2010; Kobayashi et al., 2010; Nissen et al., 1995; Preis et al., 2014). While the structures of the individual G domains or domains II-III of trGTPases superpose well, their relative orientation differs depending on the nucleotide state.

We analyzed the domain orientation in the $Ski7_C$ structures with respect to the conformations observed in canonical

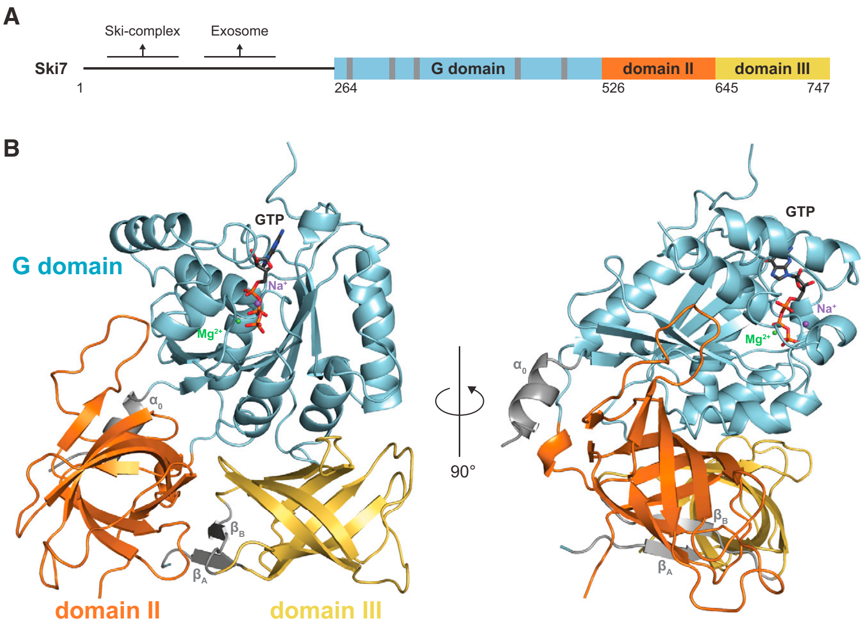

Figure 1. The Structure of the C-Terminal Domain of Ski7 Bound to GTP

(A) Schematic representation of the domain arrangement of *S. cerevisiae* Ski7. Colored rectangles highlight the C-terminal domain of Ski7 ($Ski7_C$) visualized in the structure reported here, with its three subdomains (G domain in cyan, domain II in orange, and domain III in yellow). The positions of the five conserved sequence motifs in the GTP-binding domain (G domain or domain I) are highlighted with gray bars. Regions of Ski7 that bind the Ski2-Ski3-Ski8 complex and the exosome complex are indicated.

(B) The overall structure of $Ski7_C$ bound to GTP shown in two orientations, related by a 90° anticlockwise rotation around a vertical axis. The structures are shown in cartoon representation with the G domain in cyan, domain II in orange, and domain III in yellow. GTP is shown in stick representations and the ions as spheres (green for Mg^{2+} and purple for Na^{+}). Depicted in gray are the N-terminal α helix α_0 as well as the β_A and β_B strands that are described in text. (See also Figure S1).

trGTPases. In particular, we compared $Ski7_C$ with the crystal structures of *Thermus aquaticus* EFTu, which have been determined in an active state bound to GMPPNP and tRNA (Nissen et al., 1995) and in an inactive state bound to GDP (Polekhina et al., 1996). In the EFTu structures, the G domain undergoes a dramatic three-dimensional rigid-body motion relative to domains II and III (Figure 2, right and central panel). Structural rearrangements in the so-called switch regions in response to the presence or absence of the nucleotide γ-phosphate propagate with long-range effects, resulting in the reorganization of interdomain contacts. In $Ski7_C$, domain G and domains II-III adopt a very similar architecture to that observed in the active-state snapshot of EFTu (Figure 2, left and central panels). When comparing the additional interactions with extended segments, EFTu lacks the equivalent of the N-terminal helix α_0 of $Ski7_C$ but features a similar loop that protrudes from domain II and binds the G domain as well as the 3′ end of the tRNA. We conclude that the conformation of $Ski7_C$ in both the GTP-bound and the GDP-P_i-bound structures parallels the active conformation of a canonical trGTPase.

In the active conformation, canonical trGTPases bind their cofactors at a cleft between the G domain and the barrel domains. $Ski7_C$ has a cleft at the corresponding position as the cofactor-binding surface of known trGTPases, but the detailed shape and electrostatic properties are distinct (Figure S2B). Thus, Ski7 is unlikely to use the same cofactors. Indeed, neither eRF1 nor Dom34 could augment the intrinsic GTPase activity of Ski7 in an experimental setup that robustly induces Hbs1 GTPase activity (Figure 2B).

$Ski7_C$ Can Bind Either Intact or Cleaved GTP

Superposition of the $Ski7_C$-GTP and $Ski7_C$-GDP-P_i structures shows that the guanosine moieties and the α- and β-phosphates of the two nucleotides are identically placed in the nucleotide-binding site (Figures S2B and S2C). The γ-phosphate of the GTP-bound structure and the inorganic phosphate of the GDP-P_i-bound structure are adjacent to each other, but they do not coincide. In $Ski7_C$-GTP, the γ-phosphate is connected covalently to the β-phosphate (with the canonical phosphate-phosphate distance of 2.8 Å). In $Ski7_C$-GDP-P_i, the inorganic phosphate is clearly separated from the β-phosphate of the GDP (with a phosphate-phosphate distance of 4.2 Å) (Figures 3A, 3B, and S2A).

We compared the nucleotide-binding site in the G domain of Ski7 with that of eIF5B, a trGTPase involved in subunit joining and whose G domain structure has been characterized at high resolution in several apo and nucleotide-bound states (Kuhle and Ficner, 2014a, 2014b) (Figure 3A). G domains have five consensus sequence motifs (G1–G5) (Bourne et al., 1991). Motif G1 (also known as the P loop or Walker A motif) interacts with the α- and β-phosphates of the nucleotide. Motif G2 and motif G3 (also known as the Walker B motif) bind the γ-phosphate of GTP and correspond to the switch I and switch II regions. Motifs G4 and G5 interact with the guanine base. The polypeptide backbones of the P loop, G4, and G5 motifs have essentially the same conformations in all nucleotide-bound eIF5B and $Ski7_C$ structures (Figures 3A and 3B). In the case of switch I and switch II, the polypeptide backbone in $Ski7_C$-GTP and $Ski7_C$-GDP-P_i is very similar to that observed in eIF5B-GTP and differs from eIF5B-GDP (Figure 3C). The analysis thus indicates that both $Ski7_C$ structures resemble the conformation of active GTP-bound trGTPases. In the case of the $Ski7_C$-GDP-P_i structure, the complex either mimics a putative post-hydrolysis state (e.g. before release of the P_i product and conversion to an inactive GDP-bound state) or engages the small molecules supplied at high concentrations with the crystallization buffer (i.e. GDP and inorganic phosphate) to mimic a GTP-bound state.

The Chemical Features of the Ski7 Nucleotide-Binding Site

At the sequence level, the G motifs of Ski7 contain several unusual residues at positions that are highly conserved in Hbs1,

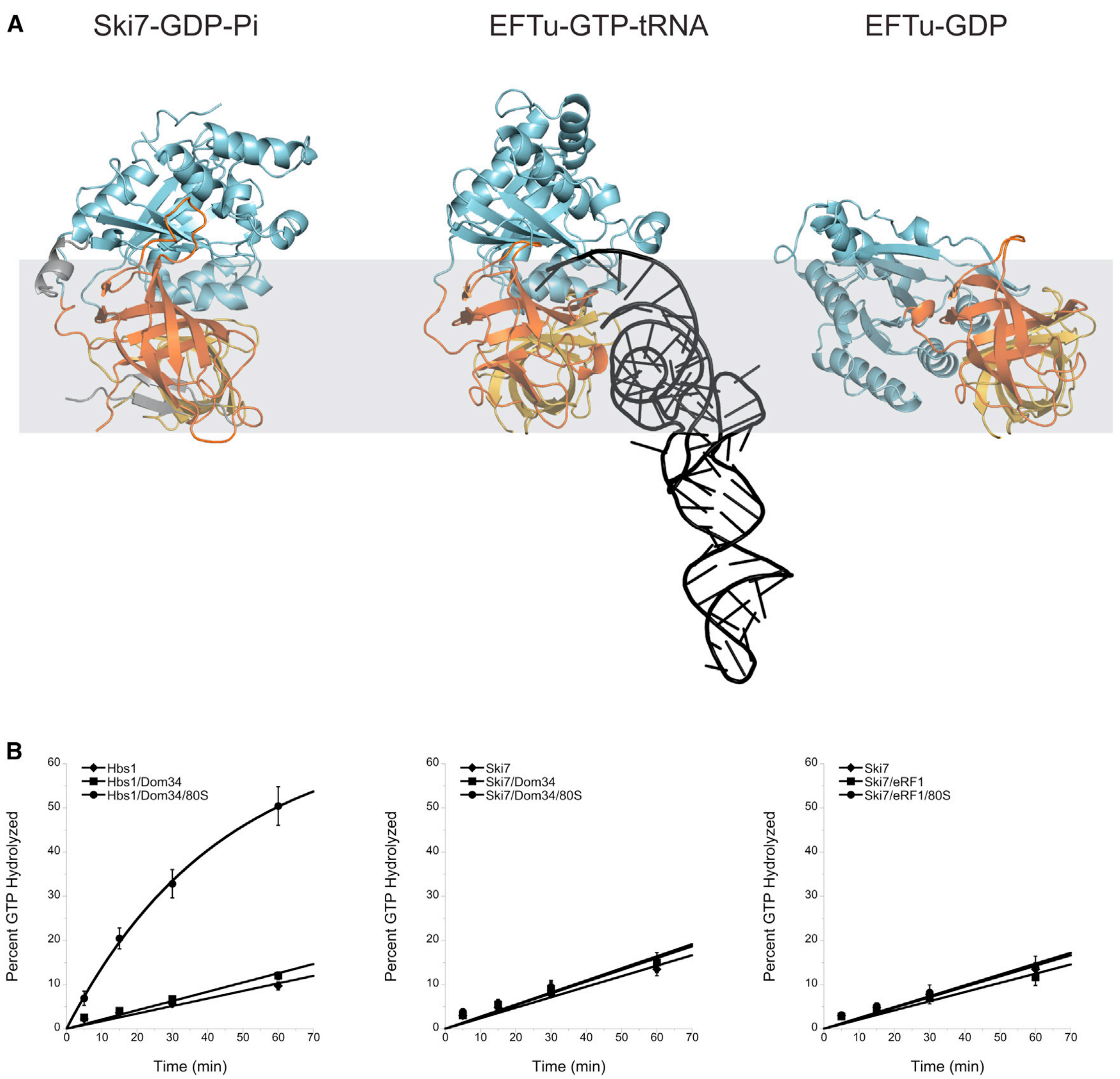

Figure 2. The Ski7$_C$ Has the Domain Architecture of an Active trGTPase

(A) The structures of Ski7$_C$-GDP-P$_i$, of EFTu-GDPPNP-tRNA (PDB: 1TTT) (Nissen et al., 1995) and of EFTu-GDP (PDB: 1TUI) (Polekhina et al., 1996) are shown after optimal superposition of domain II-III, in the same orientation and colors as in Figure 1B, right panel, RNA is shown in black.

(B) GTPase activity of Ski7 and Hbs1 in the absence and presence of co-factors and 80S ribosomes. Ski7 GTPase stimulation was not observed by Dom34, eRF1, or 80S ribosomes (middle and right panel). Hbs1 GTPase activity and stimulation by Dom34 and ribosomes was included as a positive control (left panel). The Hbs1/Dom34/80S curve fits to a single exponential, while the other curves show linear fits. Error bars are ±1 SD from the mean across three experiments. (See also Figure S2).

eRF3, EFTu, and eIF5B (Figure 4). In most cases, residues that differ from the conserved amino acids of trGTPases are nevertheless engaged in similar interactions (Figure 3A, compare right and left panels). For example, a hydrophobic residue of the G5 motif (Leu469^{Ski7}) packs against one side of the guanine base, at a position usually engaged in hydrophobic stacking interactions (Figure 3). In switch I, Ser333^{Ski7} coordinates the divalent cation and contributes to binding of the γ-phosphate, as does Gly359^{Ski7}. In switch I, Phe332^{Ski7} maintains the chemical properties of the so-called hydrophobic gate of trGTPases (Berchtold et al., 1993; Kjeldgaard et al., 1993; Villa et al., 2009). Finally, in the P loop, Asn277^{Ski7} is at the position of Asp533^{eIF5B}. In the eIF5B-GTP structure, Asp533^{eIF5B} and the main-chain carbonyl of Gly555^{eIF5B} (from switch I) coordinate a monovalent cation (either K^+ or Na^+) (Kuhle and Ficner, 2014b). The presence of a monovalent cation is thought to be a universal structural feature of active trGTPases (Kuhle and Ficner, 2014b), although it contributes little to catalysis both on and off the ribosome (Maracci et al., 2014; Åqvist and Kamerlin, 2015). In the Ski7-GTP structure, the electron density is consistent with the presence of a monovalent ion at the equivalent structural position, between Asn277^{Ski7} and the main-chain carbonyl of Gly331^{Ski7} (from switch I) (Figure 3A).

A distinguishing feature of Ski7 is the presence of Ser360^{Ski7} in switch II at the corresponding position of His598^{eIF5B}. This active site histidine residue is conserved in trGTPases, and in the presence of the ribosome and an activating cofactor it re-orients its side chain to stabilize or position the catalytic water

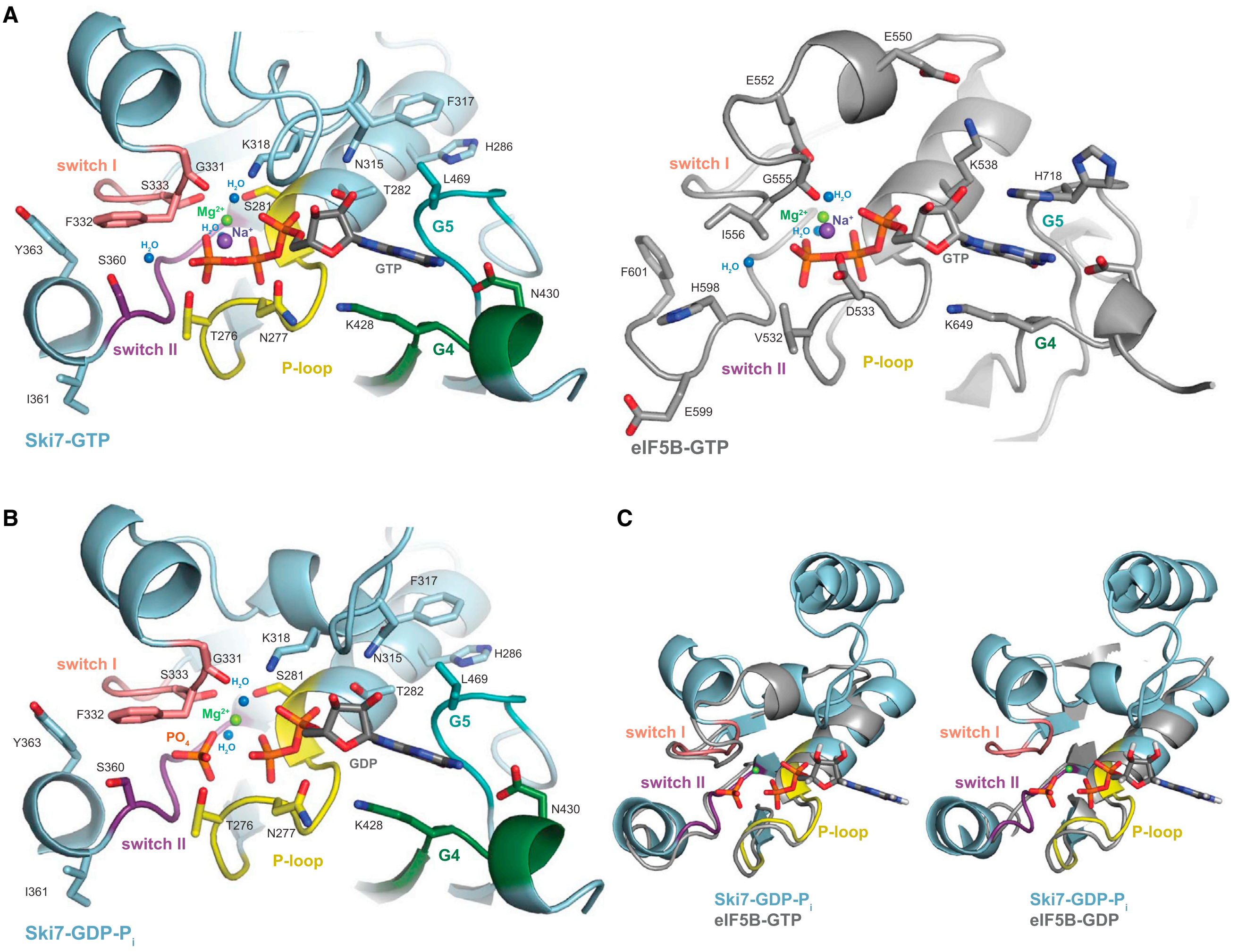

Figure 3. The Active Site of Ski7$_C$

(A) Zoom-in view of the GTP-binding site in the Ski7$_C$-GTP structure (left panel) compared with that of the trGTPase eIF5B in the active GTP-bound structure (right panel) (PDB: 4TMW) (Kuhle and Ficner, 2014b). The two molecules are shown in the same orientation after optimal superposition of their G domains. In Ski7 the five motifs of G domains are colored in yellow (P loop, also known as G1 motif), salmon (switch I, also known as G2 motif), violet (switch 2, also known as G3 motif), green (G4), and teal (G5). The Ski7 active site shows the presence of the divalent and monovalent cations and waters at the corresponding structural positions observed in eIF5B-GTP.

(B) Zoom-in view of the GTP-binding site in the Ski7$_C$-GDP-P$_i$ structure (left panel) compared in the same orientation as the molecules above.

(C) Zoom-in on the nucleotide-binding regions in the Ski7$_C$-GDP-P$_i$ structure (P loop, switch I and switch II), superposed with the corresponding regions of eIF5B in the active state (bound to GTP, PDB: 4TMW [Kuhle and Ficner, 2014b], left side) and in the inactive state (bound to GDP, PDB: 4NCL [Kuhle and Ficner, 2014a], right side). Proteins are in cartoon representation and the displayed nucleotide and ions are those bound by Ski7. The G motifs of Ski7$_C$ are colored highlighting their similarity to the active conformation of eIF5B. (See also Figure S3).

(Figures 3A and 4) (Berchtold et al., 1993; Daviter et al., 2003; Maracci et al., 2014; Villa et al., 2009). At the chemical level, it is conceivable that the function of the side chain of Ser360^{Ski7} might be to stabilize a putative catalytic water (Holliday et al., 2009) when re-oriented in the presence of the ribosome. Nevertheless, the shorter side chain would probably be suboptimal for catalysis. Another peculiarity of Ski7 is in the P loop, where Thr276^{Ski7} is at the position of Val532^{eIF5B} and engages the hydroxyl group in an unusual interaction with the nucleotide γ-phosphate (Figure 3A, left panel, and 3B). Such interaction might help to stabilize the GTP-bound state of Ski7. Analysis of Ski7$_C$ by high-performance liquid chromatography indeed indicated that the protein co-purified from *Escherichia coli* with bound GTP while purified Hbs1 did not carry any nucleotide (Figure S3A). Addition of non-cleavable nucleotide analogs during alkaline phosphatase treatment and a subsequent thermostability assay (Thermofluor) showed a decrease in the melting temperature (T_m) of GMPCP-bound Ski7C (44.0°C) in comparison with GMPPNP-bound protein (47.6°C), indicating a preference for the GTP-bound state (Figure S3B). Nucleotide-free protein showed an even lower T_m of 42.8°C and precipitated at higher concentrations.

Conclusions

trGTPases have characteristic features. At the biochemical level, they have low hydrolysis activity in isolation and require the ribosome and a specific cofactor for robust activity. At the structural

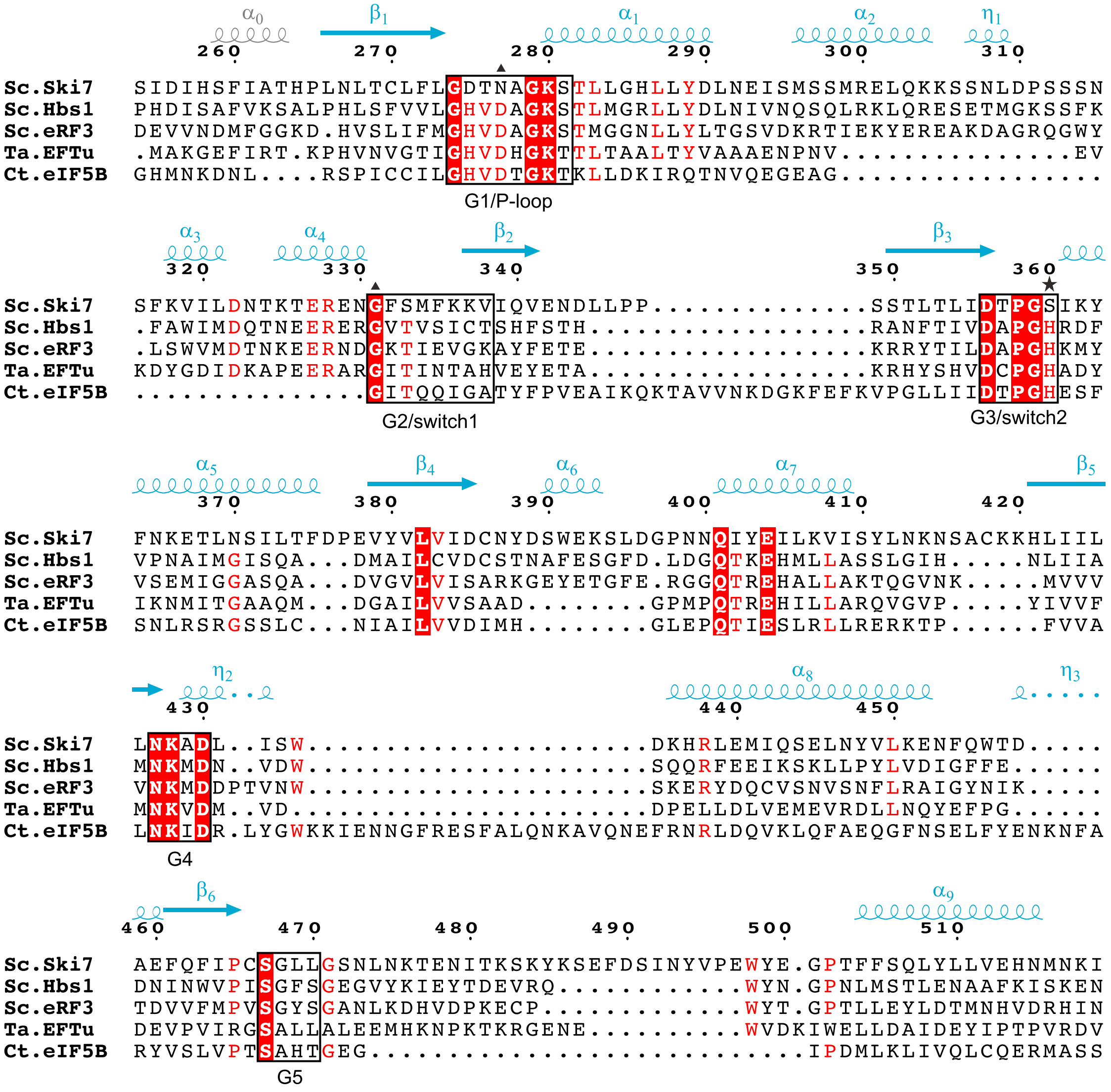

Figure 4. Structure-Based Sequence Alignment of the G Domain of Ski7$_C$ and trGTPases

The alignment includes *S. cerevisiae* (*Sc*) Ski7$_C$ G domain (residues 254–518) and the corresponding regions of *Sc* Hbs1, *Sc* eRF3, *T. aquaticus* (*Ta*) EFTu, and *Chaetomium thermophilum* (*Ct*) eIF5B. The secondary structure elements of Ski7$_C$ are shown above the sequence, colored and labeled per domain. White letters on red background share 100% of identity within the shown sequences and red letters share 80% identity. The five conserved sequence motifs of G domains (G1–G5) are highlighted in black boxes. The star indicates the position of the catalytical histidine in the trGTPases; triangles indicate the position of the monovalent metal coordinating residues.

level, they have a characteristic domain composition, a characteristic conformation in the active GTP-bound state, and a characteristic active site with a monovalent cation in addition to the universal divalent cation present in other GTPases. The work we report here indicates that the C-terminal G domain of *S. cerevisiae* Ski7 possesses the structural features of a canonical trGTPase not only in terms of domain structure, as expected from sequence analysis, but also in terms of conformation. Both the GTP-bound and the GDP-P$_i$-bound complexes of Ski7$_C$ adopt the conformation typical of trGTPases in the active GTP-bound state and show an overall similar nucleotide-binding pocket. The major differences are the presence of a different polar residue at the putative catalytic center (a serine instead of a histidine) and an additional polar residue near the γ-phosphate of GTP (a threonine instead of a valine). We envision two possible scenarios of how Ski7$_C$ might function. In one scenario, Ski7 could be a trGTPase-like protein in the full sense despite the possession of non-canonical active site residues. In this case, it would be capable of GTP hydrolysis and of switching between active and inactive conformations in the context of the ribosome and of an appropriate cofactor. Such a cofactor would be expected to function analogously to Dom34 and eRF1 but in addition would likely have to stabilize the GDP-bound form of the protein. Alternatively, in a second scenario, Ski7 could have evolved as a GTP-binding protein, i.e. as a pseudo-trGTPase. Here, a Ski7 cofactor would be expected to regulate the interaction with the ribosome with an altogether different mechanism, which does not rely on a conformational switch of the Ski7$_C$ domains.

Finally, Ski7 might even function without any cofactor. Although the biochemical data available so far would favor the concept of Ski7 as a stable GTP binder, only the identification of the cofactors that might regulate the function of the $Ski7_C$ in NSD will allow for clarification of the mechanisms.

EXPERIMENTAL PROCEDURES

Protein Expression and Purification

$Ski7_C$ (254–747) was expressed as a fusion protein with an N-terminal thioredoxin polypeptide cleavable with Prescission Protease. The expression was carried out in *E. coli* BL21 Gold pLyS cells (Stratagene) using Terrific Broth and inducing with 0.1 mM isopropyl β-D-1-thiogalactopyranoside at 18°C for 16 hr. Expression of selenomethionine-derivatized protein was carried out in minimal medium upon addition of amino acids and 50 mg/l selenomethionine prior to induction. $Ski7_C$ was purified using a Nickel affinity step (His FF, GE Healthcare) followed by cleavage of the tag with Prescission Protease, purification on heparin resin (Heparin HP, GE Healthcare) and a final size exclusion chromatography step (Superdex S200, GE Healthcare) using 20 mM HEPES (pH 7.5), 100 mM NaCl.

Crystallization and Structure Determination

$Ski7_C$ was crystallized at a concentration of 7 mg/ml with 5 mM nucleotide (GDP or GTP), 1 mM $MgCl_2$, and 5 mM Tris(2-carboxyethyl)phosphine. Crystals grew in 100 mM HEPES (pH 7.0), 700 mM NaH_2PO_4/K_2HPO_4, and 3% ethylene glycol. The crystals were cryo-protected in the presence of 25% ethylene glycol and flash-cooled in liquid nitrogen. In the case of the manganese-containing crystals, 10 mM $MnCl_2$ was included in the cryo-protectant. All X-ray diffraction data were collected at 100 K at the Swiss Light Source (SLS) synchrotron in Villigen, Switzerland. The single anomalous diffraction experiment was performed by collecting a dataset at the peak wavelength of the selenium K edge and another dataset was collected from the same crystal at the manganese K edge. The data were processed and scaled with XDS (Kabsch, 2010). The crystals belong to the space group $C222_1$ containing one molecule in the asymmetric unit. The data processing statistics are summarized in Table 1. The structure was determined and refined with standard crystallographic packages (detailed in the Supplemental Methods).

ACCESSION NUMBERS

The coordinates and structure factors of $Ski7_C$-GDP-P_i and $Ski7_C$-GTP have been deposited in the PDB with accession codes PDB: 4ZKD and 4ZKE, respectively.

SUPPLEMENTAL INFORMATION

Supplemental Information includes Supplemental Methods and three figures and can be found with this article online at http://dx.doi.org/10.1016/j.str.2015.04.018.

AUTHOR CONTRIBUTIONS

The structure was determined and analyzed by E.K. Activity assays were prepared by A.S. Experiments were performed under the direction of E.C and R.G. E.K and E.C wrote the article.

ACKNOWLEDGMENTS

We thank the Max Planck Institute of Biochemistry (MPIB) Crystallization Facility, Biophysics Facility, and the Core Facility; the beamline scientists at PXII and PXIII at SLS for assistance with data collection and members of our laboratories for useful discussions and critical reading of the manuscript. This study was supported by the Max Planck Gesellschaft, the European Commission (ERC Advanced Investigator Grant 294371 and Marie Curie ITN RNPnet) and the Deutsche Forschungsgemeinschaft (DFG SFB646, SFB1035, GRK1721, FOR1680 and CIPSM) to E.C.; by EMBO long-term fellowship, Marie Curie Actions Intra-European Fellowship (I.E.F.) and a Daimler-Benz Postdoctoral stipend to E.K.; R.G. was supported by the NIH and Howard Hughes Medical Institute.

Received: February 17, 2015
Revised: April 29, 2015
Accepted: April 29, 2015
Published: June 4, 2015

REFERENCES

Åqvist, J., and Kamerlin, S.C.L. (2015). The conformation of a catalytic loop is central to GTPase activity on the ribosome. Biochemistry *54*, 546–556.

Araki, Y., Takahashi, S., Kobayashi, T., Kajiho, H., Hoshino, S., and Katada, T. (2001). Ski7p G protein interacts with the exosome and the Ski complex for 3′-to-5′ mRNA decay in yeast. EMBO J. *20*, 4684–4693.

Atkinson, G.C., Baldauf, S.L., and Hauryliuk, V. (2008). Evolution of nonstop, no-go and nonsense-mediated mRNA decay and their termination factor-derived components. BMC Evol. Biol. *8*, 290.

Berchtold, H., Reshetnikova, L., Reiser, C.O., Schirmer, N.K., Sprinzl, M., and Hilgenfeld, R. (1993). Crystal structure of active elongation factor Tu reveals major domain rearrangements. Nature *365*, 126–132.

Bourne, H.R., Sanders, D.A., and McCormick, F. (1991). The GTPase superfamily: conserved structure and molecular mechanism. Nature *349*, 117–127.

Brown, J.T., Bai, X., and Johnson, A.W. (2000). The yeast antiviral proteins Ski2p, Ski3p, and Ski8p exist as a complex in vivo. RNA *6*, 449–457.

Chen, L., Muhlrad, D., Hauryliuk, V., Cheng, Z., Lim, M.K., Shyp, V., Parker, R., and Song, H. (2010). Structure of the Dom34-Hbs1 complex and implications for no-go decay. Nat. Struct. Mol. Biol. *17*, 1233–1240.

Daviter, T., Wieden, H.-J., and Rodnina, M.V. (2003). Essential role of histidine 84 in elongation factor Tu for the chemical step of GTP hydrolysis on the ribosome. J. Mol. Biol. *332*, 689–699.

Doma, M.K., and Parker, R. (2006). Endonucleolytic cleavage of eukaryotic mRNAs with stalls in translation elongation. Nature *440*, 561–564.

Frischmeyer, P.A., van Hoof, A., O'Donnell, K., Guerrerio, A.L., Parker, R., and Dietz, H.C. (2002). An mRNA surveillance mechanism that eliminates transcripts lacking termination codons. Science *295*, 2258–2261.

Garneau, N.L., Wilusz, J., and Wilusz, C.J. (2007). The highways and byways of mRNA decay. Nat. Rev. Mol. Cell Biol. *8*, 113–126.

Guydosh, N.R., and Green, R. (2014). Dom34 rescues ribosomes in 3′ untranslated regions. Cell *156*, 950–962.

Halbach, F., Reichelt, P., Rode, M., and Conti, E. (2013). The yeast ski complex: crystal structure and RNA channeling to the exosome complex. Cell *154*, 814–826.

Holliday, G.L., Mitchell, J.B.O., and Thornton, J.M. (2009). Understanding the functional roles of amino acid residues in enzyme catalysis. J. Mol. Biol. *390*, 560–577.

Hoshino, S.-I. (2012). Mechanism of the initiation of mRNA decay: role of eRF3 family G proteins. Wiley Interdiscip. Rev. RNA *3*, 743–757.

Houseley, J., and Tollervey, D. (2009). The many pathways of RNA degradation. Cell *136*, 763–776.

Inada, T. (2013). Quality control systems for aberrant mRNAs induced by aberrant translation elongation and termination. Biochim. Biophys. Acta *1829*, 634–642.

Januszyk, K., and Lima, C.D. (2014). The eukaryotic RNA exosome. Curr. Opin. Struct. Biol. *24*, 132–140.

Kabsch, W. (2010). XDS. Acta Crystallogr. D Biol. Crystallogr. *66*, 125–132.

Kervestin, S., and Jacobson, A. (2012). NMD: a multifaceted response to premature translational termination. Nat. Rev. Mol. Cell Biol. *13*, 700–712.

Kjeldgaard, M., Nissen, P., Thirup, S., and Nyborg, J. (1993). The crystal structure of elongation factor EF-Tu from *Thermus aquaticus* in the GTP conformation. Structure *1*, 35–50.

Klauer, A.A., and van Hoof, A. (2012). Degradation of mRNAs that lack a stop codon: a decade of nonstop progress. Wiley Interdiscip. Rev. RNA *3*, 649–660.

Kobayashi, K., Kikuno, I., Kuroha, K., Saito, K., Ito, K., Ishitani, R., Inada, T., and Nureki, O. (2010). Structural basis for mRNA surveillance by archaeal Pelota and GTP-bound EF1α complex. Proc. Natl. Acad. Sci. USA *107*, 17575–17579.

Kuhle, B., and Ficner, R. (2014a). eIF5B employs a novel domain release mechanism to catalyze ribosomal subunit joining. EMBO J. *33*, 1177–1191.

Kuhle, B., and Ficner, R. (2014b). A monovalent cation acts as structural and catalytic cofactor in translational GTPases. EMBO J. *33*, 2547–2563.

Lykke-Andersen, J., and Bennett, E.J. (2014). Protecting the proteome: eukaryotic cotranslational quality control pathways. J. Cell Biol. *204*, 467–476.

Makino, D.L., Halbach, F., and Conti, E. (2013). The RNA exosome and proteasome: common principles of degradation control. Nat. Rev. Mol. Cell Biol. *14*, 654–660.

Maracci, C., Peske, F., Dannies, E., Pohl, C., and Rodnina, M.V. (2014). Ribosome-induced tuning of GTP hydrolysis by a translational GTPase. Proc. Natl. Acad. Sci. USA *111*, 14418–14423.

Marshall, A.N., Montealegre, M.C., Jiménez-López, C., Lorenz, M.C., and van Hoof, A. (2013). Alternative splicing and subfunctionalization generates functional diversity in fungal proteomes. PLoS Genet. *9*, e1003376.

Mitchell, P., Petfalski, E., Shevchenko, A., Mann, M., and Tollervey, D. (1997). The exosome: a conserved eukaryotic RNA processing complex containing multiple 3′–>5′ exoribonucleases. Cell *91*, 457–466.

Nissen, P., Kjeldgaard, M., Thirup, S., Polekhina, G., Reshetnikova, L., Clark, B.F., and Nyborg, J. (1995). Crystal structure of the ternary complex of Phe-tRNAPhe, EF-Tu, and a GTP analog. Science *270*, 1464–1472.

Parker, R. (2012). RNA degradation in *Saccharomyces cerevisae*. Genetics *191*, 671–702.

Polekhina, G., Thirup, S., Kjeldgaard, M., Nissen, P., Lippmann, C., and Nyborg, J. (1996). Helix unwinding in the effector region of elongation factor EF-Tu-GDP. Structure *4*, 1141–1151.

Popp, M.W.-L., and Maquat, L.E. (2013). Organizing principles of mammalian nonsense-mediated mRNA decay. Annu. Rev. Genet. *47*, 139–165.

Preis, A., Heuer, A., Barrio-Garcia, C., Hauser, A., Eyler, D.E., Berninghausen, O., Green, R., Becker, T., and Beckmann, R. (2014). Cryoelectron microscopic structures of eukaryotic translation termination complexes containing eRF1-eRF3 or eRF1-ABCE1. Cell Rep. *8*, 59–65.

Rodnina, M.V. (2009). Long-range signalling in activation of the translational GTPase EF-Tu. EMBO J. *28*, 619–620.

Saito, S., Hosoda, N., and Hoshino, S.-I. (2013). The Hbs1-Dom34 protein complex functions in non-stop mRNA decay in mammalian cells. J. Biol. Chem. *288*, 17832–17843.

Schweingruber, C., Rufener, S.C., Zünd, D., Yamashita, A., and Mühlemann, O. (2013). Nonsense-mediated mRNA decay–mechanisms of substrate mRNA recognition and degradation in mammalian cells. Biochim. Biophys. Acta *1829*, 612–623.

Shoemaker, C.J., and Green, R. (2012). Translation drives mRNA quality control. Nat. Struct. Mol. Biol. *19*, 594–601.

Shoemaker, C.J., Eyler, D.E., and Green, R. (2010). Dom34:Hbs1 promotes subunit dissociation and peptidyl-tRNA drop-off to initiate no-go decay. Science *330*, 369–372.

Tsuboi, T., Kuroha, K., Kudo, K., Makino, S., Inoue, E., Kashima, I., and Inada, T. (2012). Dom34:hbs1 plays a general role in quality-control systems by dissociation of a stalled ribosome at the 3′ end of aberrant mRNA. Mol. Cell *46*, 518–529.

van Hoof, A., Staples, R.R., Baker, R.E., and Parker, R. (2000). Function of the ski4p (Csl4p) and Ski7p proteins in 3′-to-5′ degradation of mRNA. Mol. Cell. Biol. *20*, 8230–8243.

van Hoof, A., Frischmeyer, P.A., Dietz, H.C., and Parker, R. (2002). Exosome-mediated recognition and degradation of mRNAs lacking a termination codon. Science *295*, 2262–2264.

Villa, E., Sengupta, J., Trabuco, L.G., LeBarron, J., Baxter, W.T., Shaikh, T.R., Grassucci, R.A., Nissen, P., Ehrenberg, M., Schulten, K., et al. (2009). Ribosome-induced changes in elongation factor Tu conformation control GTP hydrolysis. Proc. Natl. Acad. Sci. USA *106*, 1063–1068.

Voorhees, R.M., and Ramakrishnan, V. (2013). Structural basis of the translational elongation cycle. Annu. Rev. Biochem. *82*, 203–236.

Wittinghofer, A., and Vetter, I.R. (2011). Structure-function relationships of the G domain, a canonical switch motif. Annu. Rev. Biochem. *80*, 943–971.

Zhou, J., Korostelev, A., Lancaster, L., and Noller, H.F. (2012). Crystal structures of 70S ribosomes bound to release factors RF1, RF2 and RF3. Curr. Opin. Struct. Biol. *22*, 733–742.

Structure, Volume *23*

Supplemental Information

Saccharomyces cerevisiae Ski7 Is a GTP-Binding Protein Adopting the Characteristic Conformation of Active Translational GTPases

Eva Kowalinski, Anthony Schuller, Rachel Green, and Elena Conti

SUPPLEMENTAL DATA

SUPPLEMENTAL FIGURES

FIGURE S1

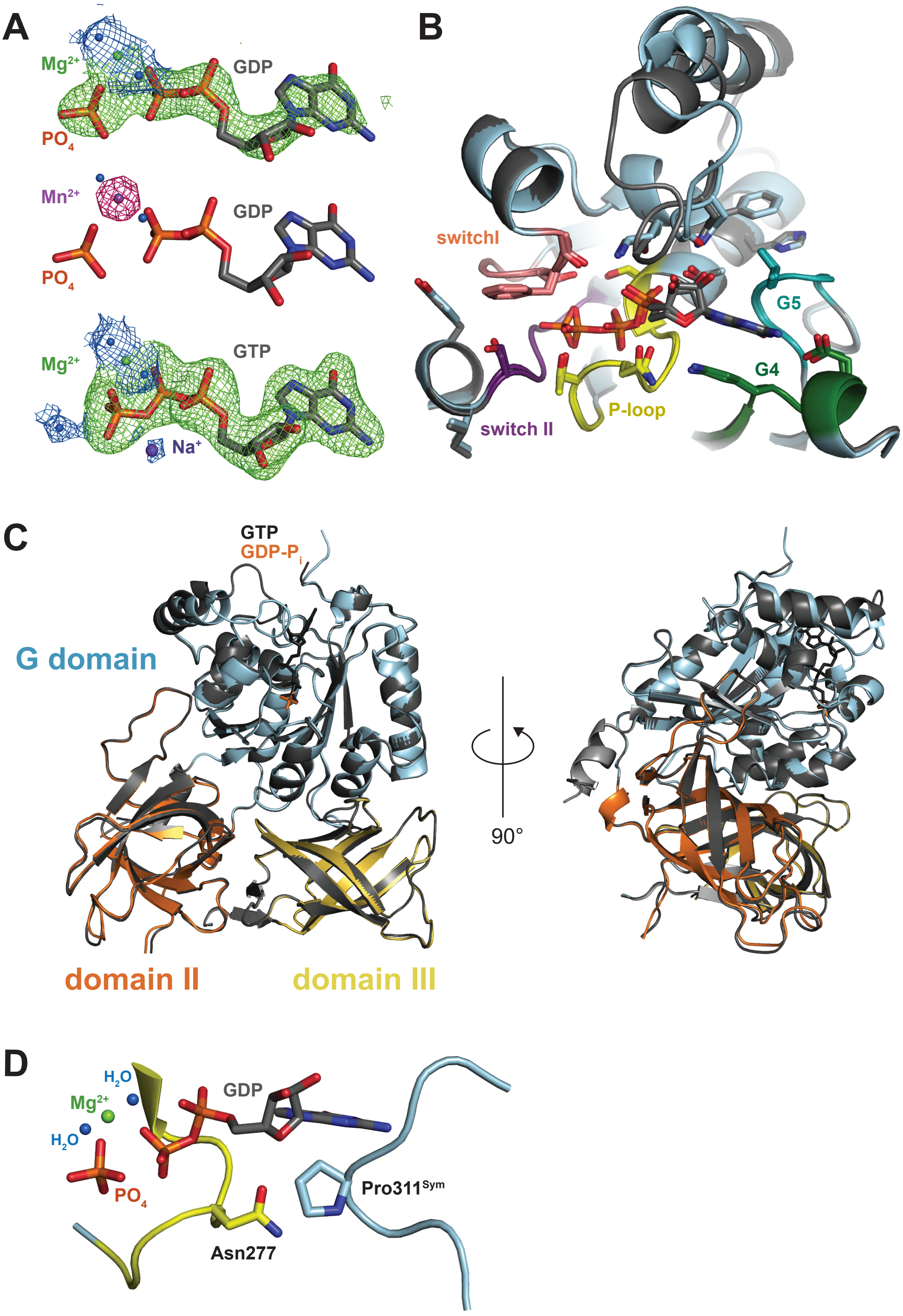

FIGURE S2

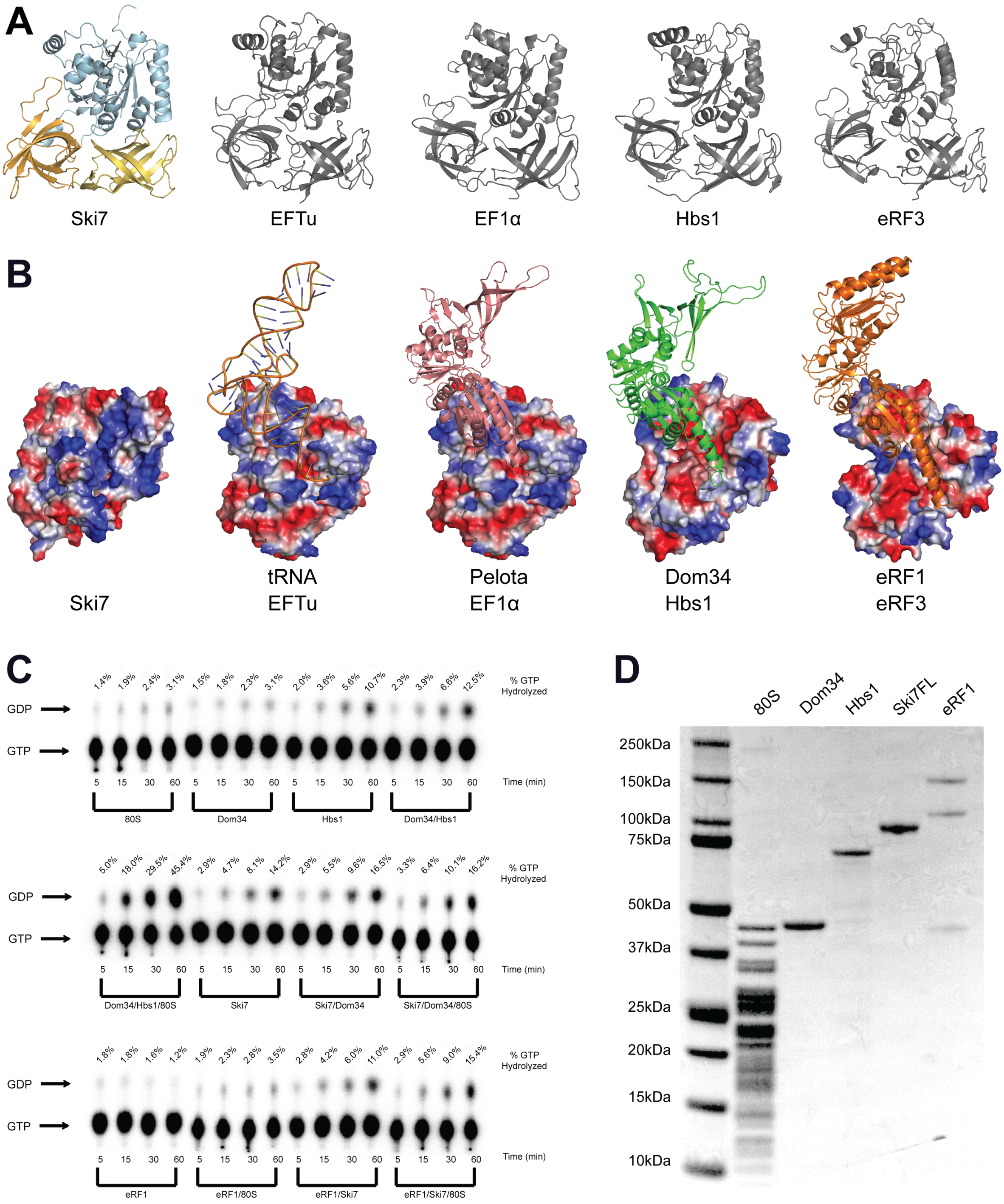
A
Ski7
EFTu
EF1α
Hbs1
eRF3
B
Ski7
tRNA
EFTu
Pelota
EF1α
Dom34
Hbs1
eRF1
eRF3
C
% GTP Hydrolyzed
GDP
GTP
Time (min)
80S
Dom34
Hbs1
Dom34/Hbs1
Dom34/Hbs1/80S
Ski7
Ski7/Dom34
Ski7/Dom34/80S
eRF1
eRF1/80S
eRF1/Ski7
eRF1/Ski7/80S
D
80S
Dom34
Hbs1
Ski7FL
eRF1
250kDa
150kDa
100kDa
75kDa
50kDa
37kDa
25kDa
20kDa
15kDa
10kDa

FIGURE S3

A

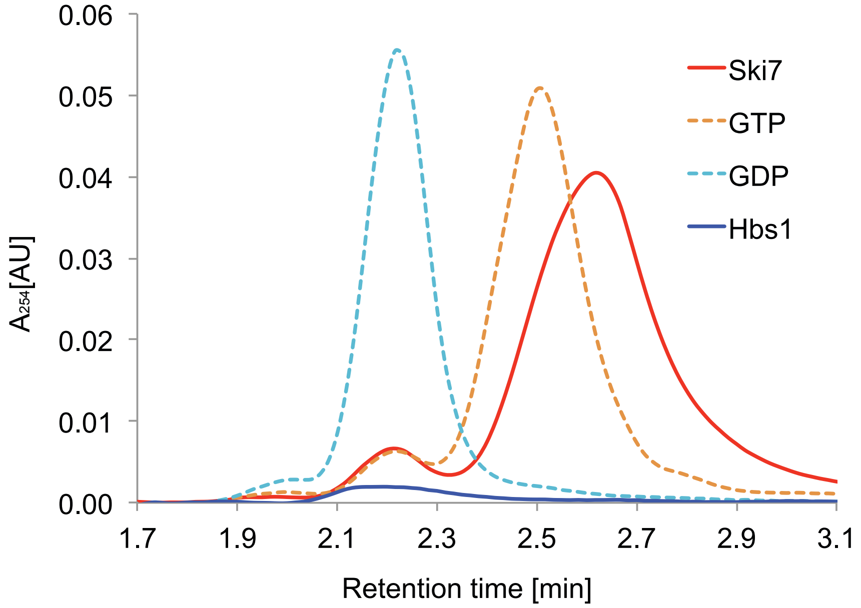

0.06
0.05
0.04
0.03
0.02
0.01
0.00
A254[AU]
1.7
1.9
2.1
2.3
2.5
2.7
2.9
3.1
Retention time [min]
Ski7
GTP
GDP
Hbs1

B

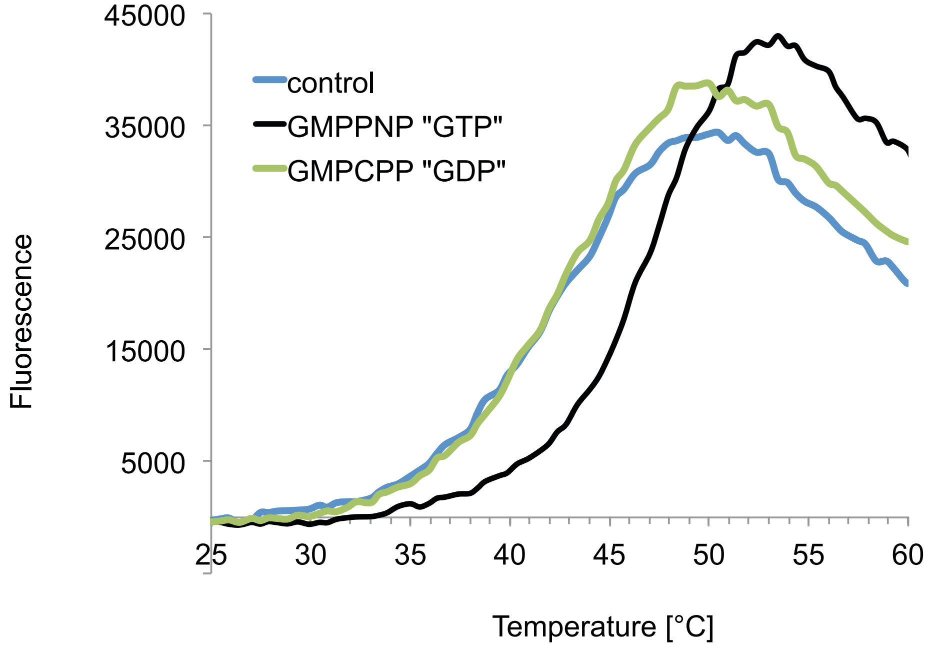

45000
35000
25000
15000
5000
Fluorescence
25
30
35
40
45
50
55
60
Temperature [°C]
control
GMPPNP "GTP"
GMPCPP "GDP"

SUPPLEMENTAL FIGURE LEGENDS

FIGURE S1 (related to Figure 1)

Detailed structural analysis of Ski7 bound to GTP and to GDP-P_i

(A) Electron density of the ligands. Upper and middle panel: Ski7$_C$-GDP-P_i, lower Ski7$_C$-GTP. GDP and P_i are shown as sticks, Mg^{2+} (green), H_2O (blue), Na^+ (purple), Mn^{2+} (purple) as spheres. 2Fo-Fc map at σ = 0.8, displayed in a radius of 1.6 Å around the Na^+ and Mg^{2+} coordinating waters in blue; Fo-Fc map at σ = 2.5 after carrying out the last refinement step omitting the nucleotide ligand and Na^+, displayed in a radius of 1.6 Å around the nucleotide, inorganic phosphate and Na^+ in green; lower panel: anomalous difference map for Manganese of a Ski7$_C$-GDP-P_i crystal that has been soaked with $MnCl_2$ prior to cryo-cooling at σ = 15.0, displayed in a radius of 1.6 Å around the Manganese ion in pink.

(B) Zoom-in view of the GTP-binding site of the superposed GTP and GDP-P_i structures. Ski7$_C$-GTP in cyan and Ski7$_C$-GDP-P_i in grey. Other colors and residues as in main Figure 4.

(C) Superposition of cartoon representations of Ski7$_C$-GTP colors as in Figure 1 and Ski7$_C$-GDP-P_i in gray shown in two orientations, related by a 90° anti-clockwise rotation around a vertical axis. GTP is shown in stick representation in black and GDP-P_i in orange.

(D) Crystal contact between Asn277 and Pro311 of a symmetry related Ski7$_C$-GDP-P_i molecule. GDP, P_i, Asn277 and Pro311are shown as sticks, Mg^{2+} (green) and H_2O (blue) as spheres.

FIGURE S2 (related to Figure 2)

The G domain and domains II-III of $Ski7_C$ are similar to those in active trGTPases, but a co-factor could not be identified

(A) The following GTPase structures were optimally aligned based on domains II and III onto the $Ski7_C$ structure and their cartoon representation is shown omitting their co-factors: EFTu (pdb code 1TTT, in complex with tRNA (Nissen et al., 1995)), EF1α (pdb code 3WXM, in complex with Pelota (Kobayashi et al., 2010)), Hbs1 (pdb code 3MCA, in complex with Dom34 (Chen et al., 2010)) and eRF3 (pdb code 4CRN, in complex with eRF1 (Preis et al., 2014)).

(B) The surface potential of different trGTPases was calculated in pymol (DeLano) and compared to Ski7 for possible co-factor binding. See Supplemental Figure 2 for the corresponding pdb accession codes.

(C) Raw data of Figure 2B. GTP hydrolysis assays with Ski7 and potential cofactors were performed using 1 μM ribosomes, 2 μM Dom34, 3 μM Hbs1, 2 μM $Ski7_{fl}$, and 2 μM eRF1 in various combinations with 1x Buffer E (20 mM Tris-HCl, pH 7.5, 100 mM KOAc pH 7.6, 2.5 mM $Mg(OAc)_2$, 2 mM DTT, 0.25 mM spermidine), 1 mM GTP, and 5 nM ^{32}P-αGTP. Reactions were quenched after 5 min, 15 min, 30 min, and 60 min with 30% formic acid. Samples were spotted on PEI-Cellulose F TLC plates (EMD Millipore) and analyzed in 0.5 M KH_2PO_4 pH 3.5. TLC plates were developed using a Typhoon FLA 9500 phosphorimaging system and quantified using ImageQuant TL (GE Healthcare Life Sciences). For the assay $Ski7_{FL}$ (1-747) was expressed as N-terminal Thioredoxin-tagged and C-terminal Strep-tagged protein using a pET-derived expression vector in E. coli BL21 Gold pLyS cells (Stratagene). Percent GTP hydrolysis is indicated above each lane.

(D) Coomassie stained gel of the proteins used in the TLC assay.

FIGURE S3 (related to Figure 3)

Nucleotide binding properties of Ski7

(A) High pressure liquid chromatography (HPLC) elution profiles with controls. Nucleotide load of $Ski7_C$ and GST-Hbs1 was analyzed at 254 absorption nm by HPLC on a RP-18-HPLC column in a buffer containing 7.5% acetonitrile, 10 mM tetrabutylammonium bromide and 100 mM KP_i buffer (pH 6.5) (Ahmadian et al., 1999). Amounts of 2 nmol nucleotides, 2 nmols $Ski7_C$ and 1.7 nmols of GST-Hbs1 were loaded.

(B) Melting curves of $Ski7_C$ after nucleotide exchange. For nucleotide exchange, 2 mg/ml of $Ski7_C$ were incubated for 2.5 h at room temperature with rSAP (NEB, 1U per 1mg of of $Ski7_C$) under addition of either no nucleotide, GMPCPP (to mimic GDP) or GMPPNP (to mimic GTP). Subsequently, 5 µl of these reactions, 35x of Sypro Orange (Invitrogen) and 45 µL of size exclusion chromatography buffer were mixed and sealed in a 96-well PCR plate (Eppendorf). In a real-time PCR system (Eppendorf) reactions were heated from 20 °C to 80 °C in increments of 0.2 °C. Fluorescence changes were monitored simultaneously. The wavelengths for excitation and emission were 470 and 550 nm, respectively. The temperature midpoint for the protein unfolding transition (Tm) was read out using the minimum of the derivative of the corresponding curve (Ericsson et al., 2006).

SUPPLEMENTAL METHODS

The program autoSHARP/SHARP was used to find 6 Selenium atom sites and to calculate phases to 3.5 Å (Vonrhein et al., 2007). An initial model was generated with Phenix Autosol (Adams et al., 2010) and the resulting electron density map was used

for automated model building in BUCCANEER (Cowtan, 2006) and manual model building with COOT (Emsley and Cowtan, 2004). The refinement was performed with Phenix Refine (Afonine et al., 2012) and the stereochemical quality was assessed by MolProbity (Davis et al., 2007). The refined coordinates of $Ski7_C$ from the GDP-P_i–bound structure were refined against the GTP-bound data, and manual building with COOT (Emsley and Cowtan, 2004) allowed modeling of the nucleotide ligand and metals (the occupancy of the Na+ ion was refined to 0.64). In the Ski7-GDP-Pi structure, no density at the corresponding position of the monovalent cation was detected, likely due to the conformation of $Asn277^{Ski7}$, whose side chain is rotated to contact a symmetry-related molecule (Figure S1D).

SUPPLEMENTAL REFERENCES

Adams, P.D., Afonine, P.V., Bunkóczi, G., Chen, V.B., Davis, I.W., Echols, N., Headd, J.J., Hung, L.W., Kapral, G.J., Grosse-Kunstleve, R.W., et al. (2010). PHENIX: a comprehensive Python-based system for macromolecular structure solution. Acta Crystallogr. D Biol. Crystallogr.

Afonine, P.V., Grosse-Kunstleve, R.W., Echols, N., Headd, J.J., Moriarty, N.W., Mustyakimov, M., Terwilliger, T.C., Urzhumtsev, A., Zwart, P.H., and Adams, P.D. (2012). Towards automated crystallographic structure refinement with phenix.refine. Acta Crystallogr. D Biol. Crystallogr. *68*, 352–367.

Ahmadian, M.R., Zor, T., Vogt, D., Kabsch, W., Selinger, Z., Wittinghofer, A., and Scheffzek, K. (1999). Guanosine triphosphatase stimulation of oncogenic Ras mutants. Proc. Natl. Acad. Sci. U.S.a. *96*, 7065–7070.

Cowtan, K. (2006). The Buccaneer software for automated model building. 1. Tracing protein chains. Acta Crystallogr. D Biol. Crystallogr. *62*, 1002–1011.

Davis, I.W., Leaver-Fay, A., Chen, V.B., Block, J.N., Kapral, G.J., Wang, X., Murray, L.W., Arendall, W.B., Snoeyink, J., Richardson, J.S., et al. (2007). MolProbity: all-atom contacts and structure validation for proteins and nucleic acids. Nucleic Acids Res. *35*, W375–W383.

DeLano, W.L. The PyMOL Molecular Graphics System; DeLano Scientific LLC: Palo Alto, CA, 2008.

Emsley, P., and Cowtan, K. (2004). Coot: model-building tools for molecular graphics. Acta Crystallogr. D Biol. Crystallogr. *60*, 2126–2132.

Ericsson, U.B., Hallberg, B.M., Detitta, G.T., Dekker, N., and Nordlund, P. (2006). Thermofluor-based high-throughput stability optimization of proteins for structural studies. Anal. Biochem. *357*, 289–298.

Vonrhein, C., Blanc, E., Roversi, P., and Bricogne, G. (2007). Automated structure solution with autoSHARP. Methods Mol. Biol. *364*, 215–230.
